# Strain Effects on Flexible Perovskite Solar Cells

**DOI:** 10.1002/advs.202304733

**Published:** 2023-10-12

**Authors:** Hongbo Liang, Wenhan Yang, Junmin Xia, Hao Gu, Xiangchuan Meng, Gege Yang, Ying Fu, Bin Wang, Hairui Cai, Yiwang Chen, Shengchun Yang, Chao Liang

**Affiliations:** ^1^ MOE Key Laboratory for Nonequilibrium Synthesis and Modulation of Condensed Matter School of Physics National Innovation Platform (Center) for Industry‐Education Integration of Energy Storage Technology Xi'an Jiaotong University Xi'an 710000 P. R. China; ^2^ State Key Laboratory of Organic Electronics and Information Displays Nanjing University of Posts and Telecommunications Nanjing 210000 China; ^3^ Joint Key Laboratory of the Ministry of Education Institute of Applied Physics and Materials Engineering University of Macau Macau 999078 P. R. China; ^4^ National Engineering Research Center for Carbohydrate Synthesis/Key Lab of Fluorine and Silicon for Energy Materials and Chemistry of Ministry of Education Jiangxi Normal University Nanchang 330000 P. R. China

**Keywords:** flexible perovskite solar cells, power conversion efficiency, stability, strain, stress

## Abstract

Flexible perovskite solar cells (f‐PSCs) as a promising power source have grabbed surging attention from academia and industry specialists by integrating with different wearable and portable electronics. With the development of low‐temperature solution preparation technology and the application of different engineering strategies, the power conversion efficiency of f‐PSCs has approached 24%. Due to the inherent properties and application scenarios of f‐PSCs, the study of strain in these devices is recognized as one of the key factors in obtaining ideal devices and promoting commercialization. The strains mainly from the change of bond and lattice volume can promote phase transformation, induce decomposition of perovskite film, decrease mechanical stability, etc. However, the effect of strain on the performance of f‐PSCs has not been systematically summarized yet. Herein, the sources of strain, evaluation methods, impacts on f‐PSCs, and the engineering strategies to modulate strain are summarized. Furthermore, the problems and future challenges in this regard are raised, and solutions and outlooks are offered. This review is dedicated to summarizing and enhancing the research into the strain of f‐PSCs to provide some new insights that can further improve the optoelectronic performance and stability of flexible devices.

## Graphical abstract

The review focuses on strain in flexible perovskite solar cells (f‐PSCs) including strain sources, strain characterization analysis, strain effects, and means of utilizing/controlling strain. The potential application scenarios of f‐PSCs and the optimization direction of high‐performance devices are summarized. The authors provide key insights and future perspectives for this important field.

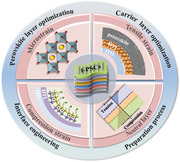

## 1 Introduction

**Figure 1:**
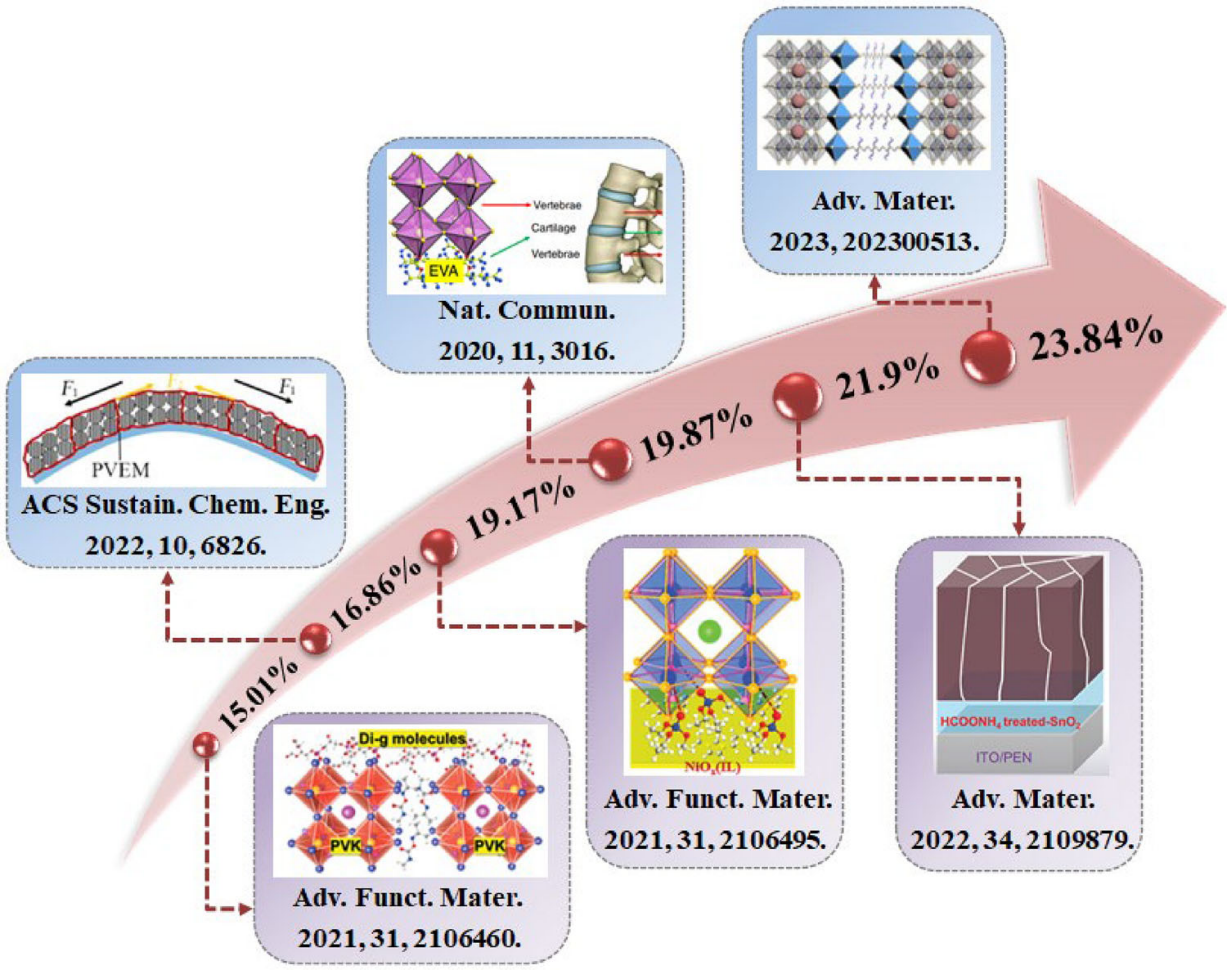
Recent research progress on strained f‐PSCs

Perovskites have attracted keen attention due to their superior semiconducting characteristics such as tunable band gap,^[^ ^1^, ^2^, ^3^ ^]^ high carrier mobility,^[^ ^4^ ^]^ high‐efficiency light absorption capability of perovskite layers,^[^ ^5^ ^]^ and low manufacturing costs.^[^ ^6^ ^]^ Combining the fabrication process, compositional, and interfacial engineering strategies, the record power conversion efficiency (PCE) of rigid perovskite solar cells (PSCs) has now reached 26%.^[^ ^7^, ^8^, ^9^, ^10^ ^]^ The excellent mechanical flexibility of perovskite materials facilitates the implementation of flexible PSCs (f‐PSCs) on a variety of flexible substrates. Owing to the advantages such as a high power‐to‐weight ratio,^[^ ^11^ ^]^ excellent flexibility,^[^ ^12^ ^]^ low processing temperature,^[^ ^13^ ^]^ and low‐cost roll‐to‐roll (RTR) manufacturing,^[^ ^14^, ^15^ ^]^ f‐PSCs hold promise to display a high commercial value in a wide range of scenarios. Benefiting from the application of engineering strategy and the development of low‐temperature preparation technology, the highest PCE of f‐PSCs has reached 23.84% (**Figure** **1**).^[^ ^16^, ^17^, ^18^, ^19^, ^20^, ^21^ ^]^ In addition to PCE, mechanical stability is also one of the important evaluation indicators for f‐PSCs.^[^ ^22^, ^23^ ^]^ The application scenarios for f‐PSCs are more flexible than for rigid devices and therefore require superior bending stability.^[^ ^24^, ^25^ ^]^ However, the strain generated by folding and bending has emerged as the most significant impediment to improving performance.^[^ ^26^, ^27^ ^]^ Certainly, strain not only occurs in the application process of the device but also in the preparation process of the semiconductor device, which may have a negative impact on the performance of the device.^[^ ^28^, ^29^ ^]^

According to the different action forms of strain, these can be classified as tensile strain, compressive strain, and microstrain. Some physical properties, such as bandgap and traps, can be manipulated indirectly by controlling the strain and thus affecting the relevant optoelectronic properties.^[^ ^30^, ^31^, ^32^, ^33^ ^]^ During the fabrication of f‐PSCs, the choice of materials and the processing of different functional layers inevitably generate strain.^[^ ^34^, ^35^ ^]^ External conditions (light, external stress, etc.) can also cause strain.^[^ ^36^, ^37^, ^38^ ^]^ Based on the effect of strain on the properties of f‐PSCs, the mechanical flexibility of f‐PSCs can be effectively improved by reducing the residual strain in perovskite films.^[^ ^39^, ^40^, ^41^, ^42^, ^43^, ^44^, ^45^, ^46^, ^47^, ^48^, ^49^ ^]^ To address the weak grain boundaries (GBs) and low crystallinity of perovskite films in f‐PSCs, Ge et al.^[^ ^41^ ^]^ introduced in situ cross‐linked elastic GBs encapsulation on the perovskite layer, which improved the elasticity and recoverability of the device. This strategy makes it easier to release stress and recover the deformation of the device and still maintains 73% of the PCE after 10 000 bending cycles. In the traditional two‐step method, porous titanium dioxide (TiO_2_) promotes the rapid conversion of lead iodide (PbI_2_) with CH_3_NH_3_I (MAI) to perovskite, but the conversion of PbI_2_ to CH_3_NH_3_PbI_3_ (MAPbI_3_) is not ideal.^[^ ^42^, ^43^ ^]^ Duan et al.^[^ ^24^ ^]^ dispersed in situ self‐polymerizing methyl methacrylate (sMMA) in PbI_2_ to form autonomous longitudinal scaffolds. The sMMA crosslinked perovskite network formed by this scaffold can release mechanical stress and improve the mechanical stability of the device. In addition to focusing on obtaining high‐quality perovskite films, passivating interfacial defects in perovskite films is also one of the effective ways to improve the strain state.^[^ ^44^, ^45^, ^46^ ^]^ Dong et al.^[^ ^47^ ^]^ developed an interpenetrating perovskite/electron transport layer (ETL) interface. This interfacial engineering strategy improves the form of interfacial interaction stresses and effectively releases the strain. Therefore, to control the influence of strain on the photoelectric properties and stability of f‐PSCs, a deep understanding of the principle of strain action is necessary and all‐important.

**Figure 2:**
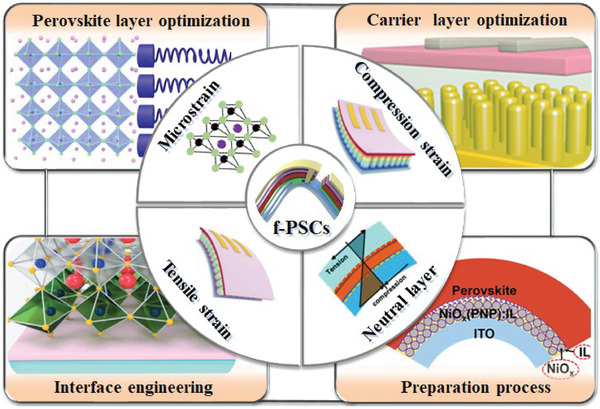
Strain classification and regulation strategies for f‐PSCs.

A systematic understanding of the sources of strain, its impact on f‐PSCs performance, and how strain can be used to improve device performance can provide new insights into f‐PSCs research and facilitate its commercialization.^[^ ^50^, ^51^ ^]^ However, there is still a lack of detailed analysis and generalization of such studies in this field. In this review, we analyze in detail the sources and effects of strain in f‐PSCs, and how to manage strain through engineering methods including interface engineering, preparation process improvement engineering, and functional layer optimization engineering (**Figure** **2**). We also outline the challenges faced by f‐PSCs, discuss methods to improve PCE, and propose perspectives for future application prospects.

## 2 Strain in f‐PSCs

### 2.1 Definition of Strain

Strain is usually defined in physics as the local relative deformation of an object caused by factors such as external force and non‐uniform temperature field.

**Figure 3:**
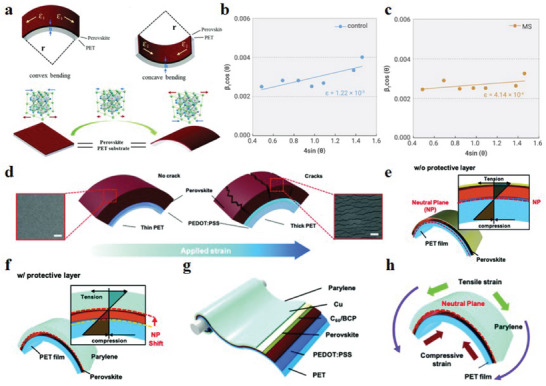
a) The upper part of the figure is a demonstration of the convex and concave bending of the flexible device. The lower part of the figure shows the lattice strain change during convex bending. Reproduced with permission.^[^ ^52^ ^]^ Copyright 2020, The Royal Society of Chemistry. Microscopic strain in b) control perovskite and c) MS‐perovskite films. Reproduced with permission.^[^ ^53^ ^]^ Copyright 2022, Elsevier. d) Schematic diagram of perovskite film changes when substrates of different thicknesses are bent. e,f) Schematic illustration of neutral layer change for f‐PSCs without and with parylene film. g) Schematic diagram of the structure of a f‐PSC based on a parylene film. h) The neutral layer located in the perovskite layer is generated by controlling the compressive and tensile strains. Reproduced with permission.^[^ ^56^ ^]^ Copyright 2019, The Royal Society of Chemistry.

At the macroscopic level, perovskite thin films generate tensile or compressive strains after being deformed by convex or concave bending, and it is accompanied by the change of lattice strain during the bending process (**Figure** **3**a).^[^ ^52^ ^]^ The strain can be calculated by the following formula

(1) $$\varepsilon_{1}=\frac{h}{2r-h}$$

(2) $$\varepsilon_{2}=\frac{h}{2r+h}$$

where *ε*_1_ is the tensile strain, *ε*_2_ is the compressive strain, *h* is the total thicknesses of the substrate and perovskite film, and *r* is the radius of curvature of the bend.

At the microscopic level, the strain can be quantitatively calculated through grazing incidence X‐ray diffraction (GIXRD) data analysis results and formulas. Specifically, there are currently two calculation methods. One is to use the Williamson–Hall equation

(3) $$\beta_{T}\cos\theta =\varepsilon \left(4\sin\theta \right)+\frac{k\lambda }{D}$$

where *β*_T_ is the full width at half maximum of the perovskite peak in X‐ray diffraction (XRD), *θ* is the diffraction angle, *k* is the shape factor, *λ* is the wavelength of the X‐ray source, *D* is the crystallite size, and *ε* is the microstrain. For example, Li et al.^[^ ^53^ ^]^ designed an ammonium methylsuccinate (MS) additive, which successfully reduced the microstrain of the perovskite film to 4.14 × 10^−4^ (Figure 3b,c). The second is to use the Stokes–Wilson equation and the Scherrer equation. In the GIXRD pattern, the half maximum (Δ2*θ*) is jointly determined by the crystal size Δ2*θ*_size_ and the lattice strain Δ2*θ*_strain_

(4) $$\Delta 2\theta \;=\;\Delta 2\theta_{\mathrm{size}}+\Delta 2\theta_{\mathrm{strain}}$$

For the convenience of calculation, the above equation can be simplified using the linear relationship between sin *θ* and Δ2*θ* × cos θ

(5) $$\Delta 2\theta \;\times \;\cos\theta =\eta \times \sin\theta +\frac{k\times \;\lambda }{D}$$

where *θ* is the diffraction peak, *η* is the lattice strain, *K* is the shape factor, *λ* is the grazing‐incidence X‐ray wavelength, and *D* is the average size of the perovskite lattice. For example, Chen et al.^[^ ^54^ ^]^ calculated the in‐plane and out‐of‐plane lattice strains at different temperatures. They found that the lattice strain of perovskite films on flexible substrates increased with increasing temperature compared with rigid substrates. However, after borax treatment, the stress is relieved to a certain extent and stabilized with the increase in temperature.

In f‐PSCs bending, if the upper layer is stretched and the lower layer is compressed, a transition surface in the cross‐section is bound to appear that is neither stretched nor compressed. The stress on this surface is almost zero. This transition surface is called the neutral surface.^[^ ^55^ ^]^ In order to explore the relationship between crack formation and strain in the perovskite layer, Lee et al.^[^ ^51^ ^]^ explored the effect of different substrate thicknesses on the crack generation of perovskite films (Figure 3d). After that, a parylene film is introduced on the perovskite film as a protective layer, which controls the formation of the neutral layer inside the perovskite and avoids the influence of the external environment on the perovskite layer (Figure 3e,f). The f‐PSCs with this idea have no cracks in the perovskite layer and exhibit ultra‐high mechanical stability (Figure 3g,h).

### 2.2 Assessment Method

Since strain can cause changes in the lattice spacing, the sample pattern obtained by XRD can be compared with the image of an unstrained (normal) device to judge the generation of strain. Tensile strain causes the spacing to increase, and compressive strain causes the spacing to decrease. If the unstrained sample is set to zero, the tensile strain is positive and the compressive strain is negative, usually expressed as a percentage. In the XRD image, if the XRD peak moves to a lower angle, it indicates tensile strain. On the contrary, if the XRD peak moves to a higher angle, it indicates compressive strain.^[^ ^57^ ^]^ However, XRD is generally used to measure large‐area local instantaneous lattice distortions, and cannot measure strains at the micron level or deep layer.

**Figure 4:**
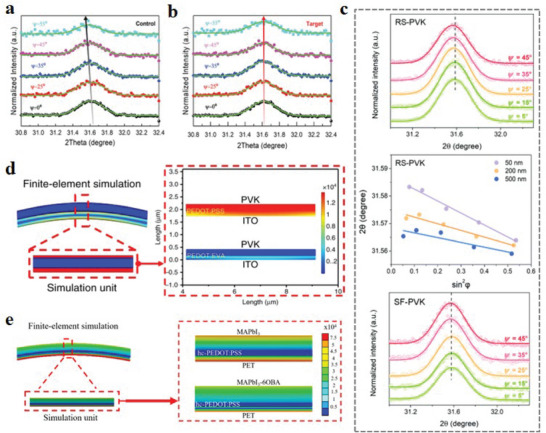
GIXRD diagrams of perovskite films at different ψ angles (from 0° to 55°): formed on SnO_2_ ETL a,b) without and with HCOONH_4_ treatment. Reproduced with permission.^[^ ^20^ ^]^ Copyright 2022, Wiley‐VCH. c) GIXRD spectra of perovskite films coated on RS‐PVK and SF‐PVK at different tilt angles at a depth of 50 nm. The middle panel shows the gradient strain of the perovskite layer. Reproduced with permission.^[^ ^59^ ^]^ Copyright 2022, The Royal Society of Chemistry. d) Schematic diagram of finite element simulation of f‐PSCs based on PEDOT:EVA HTL and PEDOT:PSS HTL. Reproduced with permission.^[^ ^19^ ^]^ Copyright 2020, Nature Publishing Group. e) FEM simulation images of f‐PSCs with or without 6OBA addition.^[^ ^68^ ^]^ Reproduced with permission. Copyright 2022, Wiley‐VCH.

Alternatively, strain can be measured by the method of GIXRD.^[^ ^58^ ^]^ At the macroscopic level, the distribution of strain with thickness in the film can be quantitatively evaluated by changing the incident angle, and the uniformity of the grains in the vertical direction can also be judged. Zheng et al.^[^ ^20^ ^]^ performed GIXRD on the (012) crystal plane of the perovskite layer on SnO_2_ ETL. As the incident angle varies from 0° to 55°, the 2*θ* parameters gradually decrease, indicating that the lattice spacing increases and the tensile strain increases (**Figure** **4**a). However, the group added with HCOONH_4_, the change of 2*θ* parameters was negligible in the same interval of angle change, indicating its response to strain release (Figure 4b). Moreover, He et al.^[^ ^59^ ^]^ proved that different flexible substrates induced different strains in perovskite films through the characterization of GIXRD. In the case of common flexible substrates, GIXRD is performed on the (012) crystal plane of the perovskite film. When the incident angle varied from 0° to 45°, the diffraction peak angle gradually became smaller, indicating the generation of tensile strain along the horizontal direction in the perovskite film. By using a special concave‐shaped polyethylene 2,6‐naphthalate (PEN)/indium tin oxide (ITO) substrate, the (012) crystal plane of the perovskite film was also subjected to GIXRD with a change of 0°–45°. There is no obvious change in the diffraction peaks, which proves that the substrate material releases the strain effectively. According to the analysis of GIXRD, it is found that strain can be adjusted in the vertical direction by reshaping the substrate (Figure 4c).

In recent reports, some researchers employ finite element method (FEM) to simulate the stress distribution in device structures.^[^ ^60^, ^61^, ^62^, ^63^, ^64^ ^]^ The FEM uses approximate mathematical methods to simulate real physical systems. Through simple and interacting elements, a finite number of unknowns is used to approximate a real system with infinite unknowns. For convenience, the FEM software is generally used for direct testing. Wang et al.^[^ ^65^ ^]^ used COMSOL Multiphysics FEM software to assess the stress distribution of the substrate when it was bent. It is found that the micro‐nano structure on the substrate can make the internal stress distribution of the polyethylene terephthalate (PET) material more uniform, and the stress is more likely to concentrate on the substrate surface. In addition to assessing the stress distribution in the device, FEM can directly quantitatively analyze the stress in the device and explore the advantage of the comparison strategy. For example, Lin et al.^[^ ^66^ ^]^ used a pressure‐assisted method to coat an Al_2_O_3_/PET protective layer on the back of the device to prepare sandwich‐structured f‐PSCs. This strategy has been proved by FEM to reduce the strain in the perovskite layer, and the flexible device exhibits excellent bending stability and environmental stability. Furthermore, the Von Mises Stress distribution in flexible devices can be explored using FEM, which can clearly describe the changes of a result in the entire model, so that the most dangerous areas in the model can be quickly determined by the analyst. Xie et al.^[^ ^67^ ^]^ calculated the stress distribution of f‐PSCs under different bending radii (*r* = 10, 5, and 2 mm) using FEM. It is found that the von Mises stress of the ITO layer is the highest in the PET‐ITO‐based device, while the maximum stress von Mises stress occurs in the Ag layer in the PET‐PH1000‐based device, and if the bend radius of the device is smaller, the von Mises stress in each layer is larger. FEM is not only employed in the device structure and the analysis of the base material but also can be used in the function layer to improve the stress distribution after the comparative analysis. Meng et al.^[^ ^19^ ^]^ used the FEM to simulate the stress distribution of f‐PSCs based on poly(3,4‐ethylenedioxythiophene):poly(ethyleneco‐vinyl acetate) (PEDOT:EVA) and poly(3,4‐ethylenedioxythiophene)‐poly(styrene sulfonate) (PEDOT:PSS). It is not difficult to find that the PEDOT:EVA interface can significantly reduce the overall stress of ITO and perovskite films (Figure 4d). In addition, Yang et al.^[^ ^68^ ^]^ simulated the stress distribution of f‐PSCs with and without a liquid crystal 4((6(acryloyloxy) hexyl)oxy)benzoic acid (6OBA) in the bent state by FEM. It is found that the stress distribution is more uniform for the perovskite films doped with 6OBA (Figure 4e).

## 3 Sources of Strain in f‐PSCs

### 3.1 Internal Generation

In conventional flexible metal halide PSCs, three groups of metal cations (Cs^+^, MA^+^, FA^+^) are matched to the structure of cubic APbI_3_ lead halide perovskite. The difference in cations radius can cause structural distortion of perovskite, especially the tilt of the B─X─B bond angle, resulting in lattice strain,^[^ ^69^, ^70^, ^71^, ^72^ ^]^ and the symmetry of the crystal structure is generally broken compared with the cubic perovskite structure. In FAPbI_3_, the larger FA cations in the 3D structure promote the tilting of the Pb‐X‐Pb Angle from the ideal 180°, resulting in lattice distortion. Phase transitions in perovskite films are diffusion‐free, and strain management can control the shape and curvature of the interface when spatial constraints arise between phases. Therefore, strain can be used to change the nano/microstructure resulting from the phase transition.^[^ ^73^ ^]^

During the nucleation and growth of perovskite films, the surface morphology of the substrate, the formation of intermediate complexes, and the influence of concentration gradients will all lead to the heterogeneous growth of the film.^[^ ^74^ ^]^ Perovskite films usually exhibit the characteristics of highly divergent grain growth directions, local strain inhomogeneity, and sub‐grain orientation disorder.^[^ ^75^, ^76^ ^]^ As a result, it is important to control the surface morphology and grain size of perovskite films to minimize the strain. Different perovskite film preparation methods are generally used to control its morphology.^[^ ^77^, ^78^ ^]^ Leonhard et al.^[^ ^79^ ^]^ used a two‐step process preparation method to deposit a mixture of MAI and MACl on PbI_2_ and successfully prepared perovskite grains with a size of a few millimeters. Adhyaksa et al.^[^ ^80^ ^]^ prepared perovskite crystallites up to 60 µm in size by dissolving Pb(CH_3_COO)_2_·3H_2_O and MABr in dimethyl sulfoxide solution by a one‐step deposition method.

**Figure 5:**
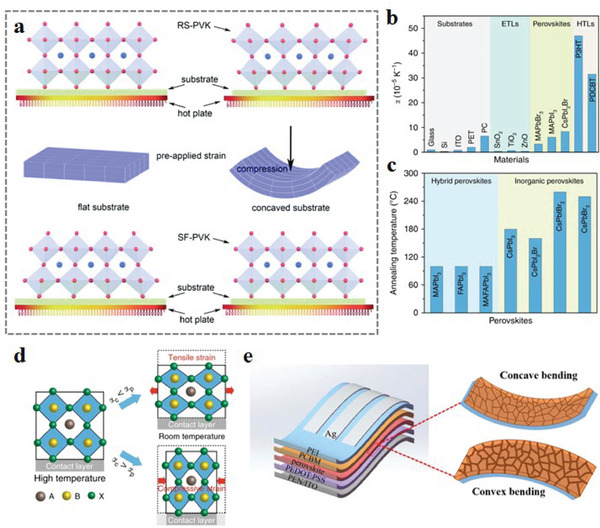
a) Schematic illustration of the formation process of residual strained perovskite films (RS‐PVK) crystallized on a substrate. The middle picture is the pre‐applied compressive strain on a concave substrate (grid is indicated to show the strain on the substrate). Strain‐free (SF‐PVK) perovskite thin films crystallized on concavely curved substrates. Reproduced with permission.^[^ ^59^ ^]^ Copyright 2022, The Royal Society of Chemistry. b) Thermal expansion coefficients of different materials for substrate, ETL, perovskite layer, and HTL in PSCs. c) Annealing temperature required for hybridization of different materials and crystallization of inorganic perovskite thin films. d) Schematic showing the formation of tensile and compressive strains. Reproduced with permission.^[^ ^85^ ^]^ Copyright 2020, Nature Publishing Group. e) Schematic diagram of the structural composition of the flexible device and its concave‐convex bending when subjected to external forces. Reproduced with permission.^[^ ^94^ ^]^ Copyright 2020, American Chemical Society.

Due to the large thermal expansion coefficient of the perovskite layer, the top of the perovskite layer will generate strain inside after cooling.^[^ ^81^, ^82^, ^83^ ^]^ As a result of the strong interfacial interaction between the perovskite layer and the substrate, compressive strain is generated at the interface during volume expansion and crystal growth of the perovskite layer, resulting in tensile strain at the top. He et al.^[^ ^59^ ^]^ pre‐applied compressive strain on the substrate material and the residual strain induced during the above‐mentioned perovskite crystal growth was released/reduced (**Figure** **5**a). It has been shown that the induced stress in the perovskite layer due to thermal coefficient mismatch is as high as 50 MPa, which is enough to induce the deformation of some softer metals.^[^ ^67^ ^]^ However, the strain caused by the difference in the thermal expansion coefficient between the perovskite layer and other adjacent layers is unavoidable (Figure 5d). Generally, some passivation materials can be selected to reduce the strain effect caused by the large difference in thermal expansion coefficient between the perovskite layer and the adjacent layer (Figure 5b).^[^ ^85^ ^]^ In addition, due to requiring high annealing temperature, inorganic PSCs generate greater interface strain than traditional organic–inorganic hybrid systems (Figure 5c). Fortunately, Xue et al.^[^ ^84^ ^]^ found that the stress of the hole transporting layer (HTL) layer is linearly related to the annealing temperature, and the increase of the HTL annealing temperature can offset the residual strain of the perovskite layer. Noteworthily, the interface strain is also related to the thickness and stiffness of the contact layer. Because the thickness of the substrate is relatively large, the substrate also plays a major role in the transfer of stress.^[^ ^86^ ^]^

### 3.2 External Influences

As for f‐PSCs, due to their bendable property, they can be used in many special scenarios such as smart wearable devices, indoor application scenarios requiring flexibility, aircraft, and aerospace.^[^ ^87^, ^88^, ^89^, ^90^, ^91^ ^]^ For example, in intelligent flexible devices that interact with human bodies, human movement is flexible and complex, resulting in unknown and variable sources of external mechanical pressure, which has strict requirements on the stability and flexibility of f‐PSCs. For the surface of metal shell structures, such as automobiles and aircrafts with uneven contact surfaces, f‐PSCs are subjected to continuous and fixed mechanical external forces on the structure. The bending resistance of f‐PSCs has a crucial impact on the life and health of users and project safety. Therefore, the most typical external influence is due to mechanical pressure, resulting in the generation of relevant strain.^[^ ^92^, ^93^ ^]^ External mechanical pressure will cause concave or convex bending of f‐PSCs (Figure 5e), which will affect the stability and lifetime of the device.^[^ ^94^ ^]^

In addition to external pressure, light, temperature, and bias voltage affect the microstrain of f‐PSCs during the operation of photovoltaic devices.^[^ ^95^, ^96^ ^]^ Under light conditions, the microstrain is enhanced since light can promote ion migration and phase separation.^[^ ^97^, ^98^ ^]^ The perovskite layer will undergo a photothermal effect under light conditions^[^ ^99^ ^]^ and the photostriction phenomenon directly converts photon energy into mechanical energy and strain.^[^ ^100^ ^]^ These can accelerate the degradation of the perovskite layer.^[^ ^101^ ^]^ Strelcov et al.^[^ ^102^ ^]^ proved that under positive/negative bias, the electric field will cause the local ion concentration of the device to change, affecting the migration of ions and causing local strain, thus the piezoelectric effect will be reduced/increased accordingly. Positive bias not only induces strain disorder but also leads to corrugated morphology on the surface of the perovskite layer, on the contrary, negative bias releases the strain and flattens the surface of the perovskite layer.^[^ ^33^ ^]^

## 4 Effect of Strain on Physical Properties in f‐PSCs

The corner‐sharing [PbI_6_]^4−^ octahedral structure changes, resulting in the film lattice structure mismatch, and strain gradient in the perovskite film.^[^ ^103^, ^104^ ^]^ Typically, the prepared perovskite films have varying degrees of internal strain in grain size, surface morphology, and grain boundary width.^[^ ^105^, ^106^ ^]^ Strain can have a significant impact on the physical properties of the perovskite layer in f‐PSCs. Here, we focus on three aspects: carrier transport, defects, and band gaps.

### 4.1 Carrier Transportation

It has been reported that carrier mobility increases with increasing compressive strain within certain limits. However, when the compressive strain is higher than the critical value, dislocations will appear, and the carrier mobility will decrease instead. Because the compressive strain can affect the energy band arrangement between the perovskite layer and the hole transport layer, the valence band maximum (VBM) is more upward than the conduction band minimum (CBM). VBM mainly consists of Pb 6s and I 5p orbitals. The interaction between these orbitals becomes stronger under compressive strain, thus pushing VBM upward, but this phenomenon requires the compressive strain size to be within a certain limit. The better energy alignment between the perovskite layer and the Fermi level of the critical layer results in higher carrier mobility.^[^ ^107^ ^]^

**Figure 6:**
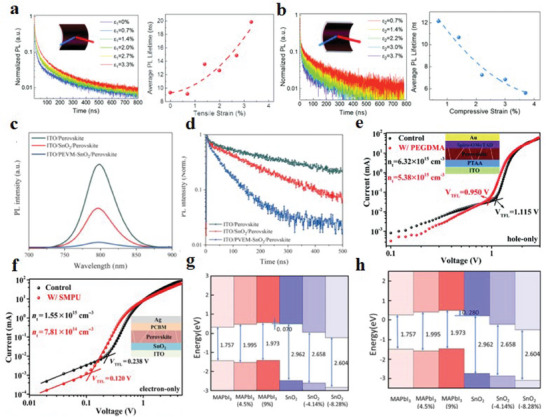
Time‐resolved PL dynamics at 760 nm for perovskite films subjected to a) tensile and b) compressive strains. Average PL lifetimes under various tensile and compressive strains. Reproduced with permission.^[^ ^52^ ^]^ Copyright 2020, The Royal Society of Chemistry. c) Steady‐PL and d) TRPL spectra of perovskite films based on ITO, SnO_2_, and PVEM‐SnO_2_. Reproduced with permission.^[^ ^17^ ^]^ Copyright 2022, American Chemical Society. e) SCLC measured dark *J*–*V* characteristics of ITO/PTAA/perovskite/Spiro‐OMeTAD/Au structured PSC hole‐only devices with or without PEGDMA addition. Reproduced with permission.^[^ ^114^ ^]^ Copyright 2022, The Royal Society of Chemistry. f) SCLC model of pure electron PSCs with ITO/SnO_2_/perovskite/PCBM/Ag structure with or without SMPU addition. Reproduced with permission.^[^ ^115^ ^]^ Copyright 2022, Wiley‐VCH. CBM and VBM: g) PbI_2_/SnO_2_ interface and h) MAI/SnO_2_ interface. Reproduced with permission.^[^ ^121^ ^]^ Copyright 2022, American Chemical Society.

In order to study the effect of strain on the carrier dynamics in perovskite thin films, Wang et al.^[^ ^55^ ^]^ used time‐correlated single photon counting to record the decay curves of the photoluminescence (PL) under different strain states. It is found that the PL lifetime increases with the increase of tensile strain and decreases with the increase of compressive strain (**Figure** **6**a,b). They concluded that tensile strain increases the carrier delocalization distance, leading to slower carrier recombination. Whereas, compressive strain reduces the lattice constant, reduces the delocalization distance, and accelerates carrier recombination. It has been demonstrated that strain affects the carrier dynamics of the hole transportation in PSCs and revealed how strain affects the band alignment between the perovskite layer and the HTL.^[^ ^107^ ^]^ Qiu et al.^[^ ^17^ ^]^ investigated the steady‐state PL and time‐resolved photoluminescence (TRPL) of glass/ITO/ETL/perovskite structures to explore the effect of poly(methyl vinyl ether‐alt‐maleic anhydride) (PVEM) additive on the carrier transport kinetics. From the PL spectral analysis, it was found that the PL intensity of glass/ITO/PVEM‐SnO_2_/perovskite was the lowest, indicating that the electron transport and extraction ability of SnO_2_ doped with PVEM was improved (Figure 6c). The average decay lifetime *τ*_avg_ of the three control samples was calculated from the TRPL spectral data. The *τ*_avg_ values of the perovskite films fabricated on glass/ITO, glass/ITO/SnO_2_, and glass/ITO/PVEM‐SnO_2_ were 451.4, 192.2, and 63.6 ns, respectively. The gradual decrease in average life confirms that the addition of PVEM to SnO_2_ increases charge‐extraction capability, corroborating the decrease in strain (Figure 6d). Combining density functional theory (DFT) calculations and Green's function, Berdiyor et al.^[^ ^108^ ^]^ demonstrated that tensile strain led to a decrease in the conductivity of the perovskite layer. However, compressive strain leads to enhanced atomic orbital overlap, increased charge transport, and improved electrical conductivity. In addition, Wang et al.^[^ ^52^ ^]^ investigated the strain‐dependent carrier dynamics in perovskite thin films and found that the PL lifetime increased with increasing tensile strain and decreased with increasing compressive strain. However, it is worth noting that there is no significant change in the transient absorption kinetics under different tensile strain conditions. This indicates that the tensile strain on the surface of the perovskite film and the compressive strain on the underlying layer compensate each other, and the internal carrier dynamics is stable as a whole.

### 4.2 Defect Properties and Non‐Radiative Recombination

Owing to the existence of inherent defects, such as interstitial halides, halide vacancies, and lead vacancies in f‐PSCs, these lead to the local strain heterogeneity of the perovskite crystal. The strain causes the formation of deep or shallow trap states, which damages the structural stability of perovskite material and leads to its degradation.^[^ ^109^, ^110^ ^]^ Using DFT to calculate the vacancy formation energy of metal halide perovskite, the formation energy increases under compression strain and decreases under tensile strain, which leads to the decrease of non‐radiative recombination and further improvement of device performance and life.^[^ ^111^, ^112^, ^113^ ^]^ The strain generated during the fabrication process affects the size of the grains and the direction of crystal growth, and the grains and sub‐grains become disordered resulting in a larger trap density.^[^ ^75^ ^]^ It has been reported that strain‐induced structural defects have a strong impact on the non‐radiative recombination of perovskite thin films in f‐PSCs, which in turn leads to a decrease in the *V*_oc_ of the device. Jones et al.^[^ ^74^ ^]^ performed TRPL and XRD measurements on the same scanned area of perovskite thin films, showing the relationship between lattice strain and defect concentration and non‐radiative recombination site increment. They found a strong inverse correlation between the charge‐carrier lifetime and the defect concentration ratio of the strained crystals. Therefore, controlling the local strain state in the device is one of the efficient ways to reduce trap density and non‐radiative recombination. Xue et al.^[^ ^114^ ^]^ fabricated hole‐only device with an ITO/poly(bis(4phenyl)(2,4,6trimethylphenyl)amine) (PTAA)/perovskite/ 2,2,7′,7′tetrakis(*N*,*N*‐di‐4methoxyphenylamine)–9,9′‐spirobifluorene (Spiro‐OMeTAD)/Au structure, and judged the effect of residual strain on the defect state density in the perovskite layer (Figure 6e). Calculated by formula

(6) $$N_{trap}=\frac{2\varepsilon_{0}\varepsilon V_{TFL}}{eL^{2}}$$

(*V*_TFL_ is the onset voltage of the trap filling limit, *e* is the elementary charge, *L* is the thickness of the active layer, *ε* is the relative permittivity of the perovskite, and *ε*_0_ is the vacuum permittivity (8.8542 × 10^−14^ F cm^−1^)), the hole trap density of PSCs with poly(ethylene glycol)dimethacrylate (PEGDMA) is 5.38 × 10^15^ cm^−3^, and that without it is 6.32 × 10^15^ cm^−3^. Similarly, in another work of their group, the defects of perovskite thin films were quantitatively estimated by space‐charge‐limited current (SCLC). Using the above formula (Figure 6f), the trap densities of perovskite films with and without shape memory polyurethane (SMPU) are 7.18 × 10^14^ and 1.55 × 10^15^ cm^−3^.^[^ ^115^ ^]^ Therefore, releasing the residual strain can effectively reduce the trap density in perovskite films.

### 4.3 Bandgap

In the band structure of halide perovskite, the valence band is controlled by the antibonding overlap of the Pb s orbitals and the halide p orbital, and the conduction band is mainly controlled by the non‐bonding overlap between Pb p orbitals with minor contributions from halide p orbitals.^[^ ^116^, ^117^, ^118^ ^]^ As mentioned above, the strain will cause a change in B─X bond length and B─X─B bond angle, and correspondingly, the change of orbital overlap will lead to a change in band dispersion.^[^ ^119^, ^120^ ^]^ Pu et al.^[^ ^121^ ^]^ studied the CMB and VBM of the perovskite layer and the SnO_2_ ETL boundary under the influence of different strains, and obtained two different band alignments of the SnO_2_/perovskite interface by using the Perdew–Burkes–Ernzerhof (PBE)–Heyd–Scuseria–Ernzerhof (HSE) density functional method. At the PbI_2_/SnO_2_ and MAI/SnO_2_ interfaces, the CBM of perovskite increases with increasing tensile strain, and the CBM of SnO_2_ decreases in band structure with increasing compressive strain (Figure 6g,h). The CBM difference between the perovskite and SnO_2_ is negligible when the strain mainly occurs in the perovskite layer, while the CBM difference between the two layers is enlarged when the SnO_2_ is mainly subjected to strain.^[^ ^122^, ^123^ ^]^ Furthermore, Chen et al.^[^ ^107^ ^]^ explored the bandgap change of α‐FAPbI_3_ under the influence of compressive strain by Raman spectroscopy and PL spectroscopy. With increasing compressive strain, the Raman signal is stronger and more distinguishable, the light absorption in the visible region is red‐shifted, and the absorbance is stronger than the control one. In a word, strain can cause tilting, shrinkage, or expansion of the [PbI_6_]^4‐^ octahedron, and the bandgap increases with tensile strain and decreases with compressive strain.^[^ ^33^, ^108^ ^]^ Incidentally, the bandgap of low‐dimensional (LD) perovskites is also affected by strain. For example, the relationship between the bandgap and strain of 1D perovskite is parabolic, and the bandgap of 2D perovskite widens with increasing tensile strain and narrows with increasing compressive strain.^[^ ^124^, ^125^ ^]^

## 5 Strategy to Alleviate Strain in f‐PSCs

The strain has an important impact on the performance and stability of f‐PSCs, and there are currently many strategies to control the strain in devices. Overall, they can be classified into three categories: the preparation process, functional layer optimization (**Tables** **1** and **2**), and interface engineering (**Table** **3**).

**Table 1:** Methods to improve the key performance of f‐PSCs by controlling strain of functional layers (carrier transport layer).

Functional layer	Additives/materials	Structural composition of f‐PSCs	PCE of f‐PSCs	Bending stability	Ref.
ETL	HCOONH_4_	PEN/ITO/HCOONH_4_ and SnO_2_/Perovskite/Spiro‐OMeTAD/Au	21.90%	4000 cycles, 90%	[20]
ETL	PVEM	PEN/ITO/SnO_2_ w/o PVEM/Perovskite/Spiro‐OMeTAD/Ag	16.86%	5 mm, 40 cycles, 92%	[17]
ETL	ZnO@ZnS NRs	ITO/PET/ZnO@ZnS NRs /Perovskite/Spiro‐OMeTAD/Ag	14.68%	/	[152]
ETL	ZnO NW array	PEN/ITO/ZnO NW array/ Perovskite/Spiro‐OMeTAD/Au	12.80%	/	[153]
ETL	F‐TiO*_x_*‐SnO_2_	ITO/PET/F‐TiO*_x_*‐SnO_2_/ Perovskite/Spiro‐OMeTAD/Ag	22.70%	5 mm, 4000 cycles, 82.11%	[200]
ETL	3AAH^a)^	ITO/SnO_2_/Perovskite/Spiro‐OMeTAD/Ag	23.36%	5 mm, 4000 cycles, 84%	[203]
HTL	NiOx NaPAs	PET/ITO/NiO*_x_* “50‐CL+100‐NaPA”/ Perovskite/PC61BM/BCP/Ag	17.23%	10 mm, 500 cycles, 80%	[155]
HTL	PEDOT:PTP	PET/ITO/PEDOT:PTP/Perovskite /PC61BM/BCP/Au	12.68%	7 mm, 1500 cycles, 92%	[158]
HTL	NC‐PEDOT:PSS	PEN/ITO/NC‐PEDOT:PSS/Perovskite /PC61BM/BCP/Ag	12.32%	2 mm, 1000 cycles, 95%	[159]

^a)^ 3‐Aminopropionic acid hydroiodide.

**Table 2:** Methods to improve the key performance of f‐PSCs by controlling strain of functional layers (perovskite layer).

Functional layer	Additives/materials	Structural composition of f‐PSCs	PCE of f‐PSCs	Bending stability	Ref.
Perovskite	SBS‐Pu^a)^	PDMS/PEDOT:PSS/Perovskite/PC61BM/PEI/PEDOT:PSS/PDMS	15.01%	2 mm, 5000 cycles, 80%	[180]
Perovskite	PEGDMA	PEN/ITO/SnO_2_/Perovskite/Spiro‐OMeTAD/Au	21.41%	5 mm, 5000 cycles, 86%	[114]
Perovskite	Methylammonium succinate	PEN/ITO/SnO_2_/Perovskite/Spiro‐OMeTAD/Au	22.50%	6 mm, 10 000 cycles, 85%	[53]
Perovskite	Borax	PEN/ITO/SnO_2_/Perovskite/Spiro‐OMeTAD/MoO_3_/Ag	21.63%	5 mm, 10 000 cycles, 91.8%	[54]
Perovskite	OETC^b)^	PET/ITO/SnO_2_/Perovskite/Spiro‐OMeTAD/Au	23.40%	5 mm, 5000 cycles, 90%	[33]
Perovskite	TA‐Ni^c)^	PET/ITO/SnO_2_/Perovskite/Spiro‐OMeTAD/Au	23.68%	5 mm, 20 000 cycles, 91%	[21]
Perovskite	PNPs^d)^	PET/ITO/SnO_2_/ PNPs@NiO_x_@PAM/Spiro‐OMeTAD/Au	16.57%	120°, 1000 cycles, 80%	[202]
Perovskite	Sodium hyaluronate	PEN/ITO/PTAA/Perovskite /PCBM/BCP/Ag	20.01%	2 mm, 6000 cycles, 90%	[165]
Perovskite	Monomer ETPTA	PEN/ITO/TFB/Perovskite /PCBM/BCP/Ag	13.60%	10 mm, 2000 cycles, 80%	[166]
Perovskite	SMPU	PEN/ITO/SnO_2_/Perovskite/Spiro‐OMeTAD/Au	21.33%	8 mm, 6000 cycles, 80%	[115]
Perovskite	Sulfonated graphene oxide	PEN/hc‐PEDOT:PSS/NiO_x_/Perovskite /PCBM/BCP/Ag	20.56%	3 mm, 1000 cycles, 80%	[173]
Perovskite	6OBA	PEN/hc‐PEDOT:PSS/NiO_x_/ Perovskite/PCBM/Ag	19.87%	3 mm, 1000 cycles, 88%	[68]
Perovskite	g‐C_3_N_4_	PDMS/hc‐PEDOT:PSS/PEDOT: PSS/Perovskite/C60/BCP/Ag	8.56%	3 mm, 600 cycles, 92%	[185]

^a)^ poly(styrene‐*co*‐butadiene)‐polyurethane;

^b)^ bis((3‐methyloxetan‐3‐yl) methyl)thiophene‐2,5‐dicarboxylate;

^c)^ a crosslinkable monomer (5‐(1,2‐dithiolan‐3‐yl)pentanehydrazide hydroiodide;

^d)^ Perovskite nanopillars.

**Table 3:** Reports of strain control by interface engineering.

Interface material	Application location	Structural composition of f‐PSCs	PCE of f‐PSCs	Bending stability	Ref.
Diphosphatidyl‐glycerol	Perovskite/ETL	PDMS/PEN/hc‐PEDOT:PSS/PEDOT:PSS/Perovskite/Di‐g/PCBM/BCP/Ag/PDMS	20%	3 mm, 10 000 cycles, 85%	[16]
Bio‐IL	HTL/Perovskite	PEN/ITO/Bio‐IL/Perovskite/BCP/Ag	21.08%	3 mm, 2000 cycles, 90%	[195]
PEDOT:EVA	HTL/Perovskite	PEN/ITO/PEDOT:EVA /Perovskite/PCNM:BCP/Ag	19.87%	3 mm, 7000 cycles, 85%	[19]
Self‐assembled monolayer	HTL/Perovskite	PEN/ITO/SnO_2_/SAM/3D‐MHP/LD‐MHP/Spiro‐OMeTAD/Au	21.03%	5 mm, 10 000 cycles, 88%	[196]
PASCA‐Br	ETL/Perovskite	FTO/TiO_2_/PASCA‐Br/Perovskite/Spiro‐OMeTAD/MoO_3_/Ag	17.45%	/	[83]
Y6	ETL/Perovskite	PEN/ITO/SnO_2_/Y6/Perovskite/Spiro‐OMeTAD/MoO_3_/Au	20.09%	8 mm, 1000 cycles, 80%	[198]
PTCDI^a)^	Perovskite/ETL	PET/PEDOT:PSS/Perovskite/PTCDI/ Cr_2_O_3_/Cr/Au	12%	/	[11]
PenAAc	HTL/Perovskite	PEN/ITO/PTAA/PenAAc/Perovskite/C60/BCP/Ag	21.52%	4 mm, 5000 cycles, 91%	[199]
LCE^b)^	ETL/Perovskite	PEN/ITO/SnO_2_/LCE/Perovskite/Spiro‐OMeTAD/Ag	22.10%	4 mm, 5000 cycles, 86%	[201]

^a)^ *N*, *N*′‐dimethyl‐3,4,9,10‐tetracarboxylic perylene diimide;

^b)^ Liquid crystalline elastomers.

### 5.1 Preparation Process

**Figure 7:**
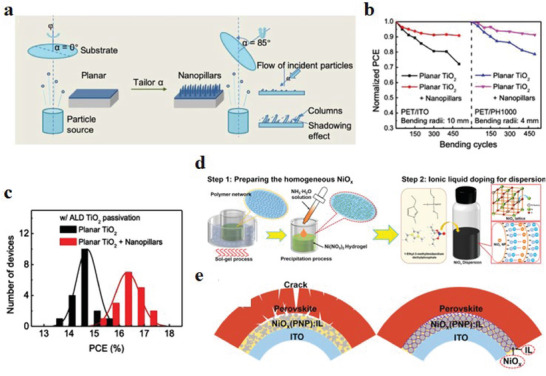
a) Schematic diagram of the preparation process of TiO_2_ and TiO_2_ nanocolumns by GLAD method. b) Normalized PCE of f‐PSCs based on planar TiO_2_ and planar TiO_2_ nanopillars after bending cycles (10 and 4 mm bending radius). c) PCE histograms of 18 TiO_2_ (15 nm) (black) and TiO_2_ (15 nm) integrated nanopillar (100 nm) (red) PSCs passivated by ALD TiO_2_. Reproduced with permission.^[^ ^130^ ^]^ Copyright 2020, Wiley‐VCH. d) Schematic illustration of the preparation process for obtaining high crystallinity and well‐dispersed NiO*_x_* colloidal solutions. Comparison of strain effects in f‐PSCs based on e) NiO*_x_* and f) NiO*_x_* (PNP):IL HTL. Reproduced with permission.^[^ ^18^ ^]^ Copyright 2021, Wiley‐VCH.

As mentioned above, thermal expansion coefficient mismatch and annealing treatment caused the strain during the preparation process. The corresponding adjustment of the preparation technology is an important way to control this strain.^[^ ^126^, ^127^, ^128^ ^]^ Zhao et al.^[^ ^129^ ^]^ did not use the traditional high‐temperature annealing process, but grew the MAPbI_3_ perovskite film at room temperature in a vacuum atmosphere for 3 days. The authors found no stress in these films, proving that temperature is the key factor for the lattice strain in the perovskite layer. Although lowering the annealing temperature can reduce the tensile strain in the perovskite film, the low quality of the resulting perovskite film also leads to the degradation of device performance. Normally, high‐temperature annealing affects not only the strain in the perovskite film but also the strain in the electron ETL.^[^ ^86^ ^]^ Wu et al.^[^ ^130^ ^]^ innovatively used the low‐temperature glancing angle deposition (GLAD) method to deposit TiO_2_ nanocolumn arrays on flexible substrates, effectively avoiding the impact of high‐temperature annealing on the performance of f‐PSCs (**Figure** **7**a). The TiO_2_ nanopillars generated using this method provide more adhesion sites for the perovskite layer, reducing the stress distribution and improving the interfacial adhesion. Compared with the TiO_2_ ETL on rigid substrates, in perovskite solar devices made of PET/ITO substrates, the bending radius can reach 4 mm, which significantly improves the flexibility of the devices, and in the PET/PH1000‐based device, more than 90% of the initial PCE was maintained after bending 450 cycles under 4 mm (Figure 7b). The average PCE of PSCs based on TiO_2_ nanopillars is 16.33%, which is about 1.6% higher than that of the common method (Figure 7c). Among the materials that can be used as HTL, NiO*_x_* has attracted much attention because it can be simply prepared at low temperatures and low cost.^[^ ^131^, ^132^ ^]^ Zhang et al.^[^ ^18^ ^]^ successfully prepared NiO*_x_* nanomaterials with high dispersibility and crystallinity by using a polymer network that can be realized by the sol–gel method. After dispersing NiO*_x_* nanoparticles (NPs) in the solution, 1‐ethyl‐3‐methylimidazole diethyl phosphate ionic liquid (IL) was added as a surfactant to obtain a NiO*_x_* colloidal solution (Figure 7d). Flexible perovskite devices based on NiO*_x_*:IL solution showed no significant cracking after bending 5000 times, in contrast to devices made with normal NiO*_x_* layers where the perovskite films showed significant cracking. The high‐viscosity ionic liquid enhances the interfacial connection between the perovskite layer and the HTL, reducing stress generation and enhancing the stability of the device (Figure 7e,f). The PCE of the PSCs devices made using this HTL material was 20.92% and up to 19.17% for f‐PSCs, providing excellent environmental stability. In addition to the above methods, vapor deposition and inkjet printing are also effective ways to obtain low‐strain gradient functional layers.^[^ ^133^, ^134^, ^135^ ^]^

The ion migration problem in perovskite thin films is one of the obstacles to its commercial application, and it is particularly important to explore the relationship between the strain relaxation effect and ion migration. In order to investigate the nature of ion migration in the perovskite films, Meng et al.^[^ ^81^ ^]^ determined the total ionic conductivity by Jonscher's law, and tested its relationship with strain. They found that the ionic conductivity of the unstrained film was 3 × 10^−4^ S m^−1^, while the strained film provided a relatively high conductivity of 5 × 10^−4^ S m^−1^. They went on to investigate the trap density using SCLC. The calculated trap densities are 1.54 × 10^16^ and 6.62 × 10^15^ cm^−3^ for perovskite thin film with and without strain, respectively. This indicates a higher density of traps in strained perovskite films. Therefore, strain‐induced defects play a key role in causing degradation by accelerating ion migration. Strain release slows ion migration and improves device stability. Ion migration can help relax tensile strain by moving ions toward the strained region. But when ion migration is hindered, strain is created at the GBs.^[^ ^85^ ^]^ Zhao et al.^[^ ^129^ ^]^ investigated the effect of strain on the stability of MAPbI_3_ thin films. They found that the mechanism of strain‐accelerated degradation was related to ion migration. The perovskite films with larger strains have smaller activation energy for ion migration both under dark and light conditions. Ion migration is faster in strained perovskite films. MAPbI_3_ degrades to PbI_2_ faster because MA^+^ and I^−^ ions can more easily migrate from the MAPbI_3_ film to produce PbI_2_. The increase in ion migration at larger lattice strains can be explained by the additional driving force, where the ion migration process relaxes the lattice strain, thereby reducing the free energy of the system. From this point of view, controlling the strain of perovskite films generated during the high‐temperature annealing process is an effective way to control ion migration and enhance stability in f‐PSCs. Furthermore, in 2D perovskites, the ion mobility is suppressed by adjusting the process to improve the cation packing mode, relieve the microstrain, and reduce the electron–phonon (E–P) interaction.^[^ ^136^, ^137^ ^]^

### 5.2 Function Layer Optimization

#### 5.2.1 Substrate

**Figure 8:**
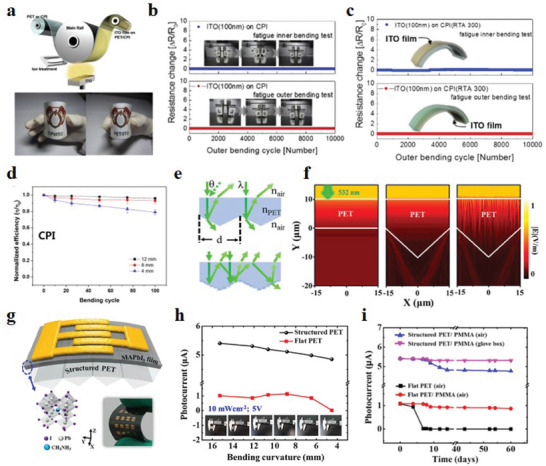
a) Demonstration diagram of the RTR sputtering process for flexible ITO thin films prepared on CPI substrates. Physical pictures of flexible ITO/CPI and ITO/PET samples in the lower part. Dynamic internal and external bending fatigue test results of b) as‐deposited ITO/CPI and c) annealed ITO/CPI substrates. d) Normalized PCE versus number of bending cycles for CPI‐based f‐PSCs. Reproduced with permission.^[^ ^139^ ^]^ Copyright 2016, Elsevier. e) Demonstration of the ray path of light in different structured PET substrates. f) Simulation diagram of near‐field electric field distribution: planar PET, structured PET, and structured PET with micron‐sized scatterers on the surface. g) Schematic diagram of Au/MAPbI_3_/structured PET substrate structured FPD. The lower left inset is a schematic diagram of the 3D structure of MAPbI_3_, and the lower right inset is an optical image of the flexible device. h) Performance comparison of perovskite FPDs on different substrates under different bending times and different curvature radii. i) Stability comparison of devices with different structures. Reproduced with permission.^[^ ^65^ ^]^ Copyright 2020, American Chemical Society.

Compared with the rigid substrate, the difference in thermal expansion coefficient between the flexible substrate and perovskite layer is smaller, but the roughness of the flexible substrate material can affect the growth of the perovskite layer in particular.^[^ ^85^ ^]^ Flexible substrate materials can be classified into plastics, metal materials, etc. Plastic substrates in most cases cannot withstand the high‐temperature annealing process required to manufacture metal oxide carrier transporting layers. However, the high temperature‐resistant flexible metal foil has the problem of poor light transmittance when the thickness is large and low conductivity when the thickness is small.^[^ ^138^ ^]^ As mentioned above, the difference in flexible substrate materials can also have an effect on the generation of strain distribution in the device. Park et al.^[^ ^139^ ^]^ prepared a new type of transparent electrode for f‐PSCs using the RTR process by sputtering ITO films on colorless PI (CPI) and PET substrates (**Figure** **8**a). Through the characterization study of f‐PSCs based on two different electrodes, it was found that the PCE of CPI/ITO‐based solar cells was slightly higher than that of PET/ITO‐based f‐PSCs. After bending 100 times under different bending radii (4, 8, and 12 mm), CPI/ITO‐based devices keep more than 80% of the initial PCE (Figure 8d). The authors also performed dynamic fatigue tests on f‐PSCs with a bending radius of 5 mm and found that the as‐deposited and annealed ITO/CPI samples showed no change in electrical resistance (DR) after 10 000 bending cycles (Figure 8b,c). This is attributed to the excellent thermal stability and mechanical flexibility of the CPI substrate which reduces strain during internal and external bending, and the structured design of flexible perovskite optoelectronic device substrates also shows obvious potential for improving the near‐field optics and stress distribution of the devices. Wang et al.^[^ ^65^ ^]^ fabricated the periodic micro/nanostructures on PET substrates using femtosecond laser direct writing (FsLDW) technology, and the effect of the structure on the photoresponse of photoactive materials was explored systematically. Lumerical software was used to simulate the near‐field electric field distribution on the surface of different structures, and it was found that the micro‐nano structure would locally enhance the near‐field electric field with a significant increase in light scattering and trapping ability (Figure 8e,f). Flexible perovskite photodetectors (FPDs) were fabricated on structured PET substrates, and the influence of different substrate structures on the stability of device performance was explored (Figure 8g). The comparison found that the internal stress distribution of the PET substrate with structured micro/nanostructures was more uniform, and the mechanical stability of the device was also better than the control one (Figure 8h,i). This logical strategy can obviously also be applied to f‐PSCs. In addition to the research on new substrate materials and the modification of substrate materials, the physical parameters of substrate materials also have an important impact on the performance of f‐PSCs. Lee et al.^[^ ^56^ ^]^ tested the performance of f‐PSCs by varying the thickness of the substrate to confirm the effect of strain. The results of the experiments show that the performance of the devices is highly dependent on the overall crack density of the perovskite films. The appearance and density of cracks in perovskite films depend on the thickness of the substrate and the bending radius. Therefore, an appropriate substrate thickness can regulate the neutral plane position and release the residual strain.

#### 5.2.2 Carrier Transporting Layer

**Figure 9:**
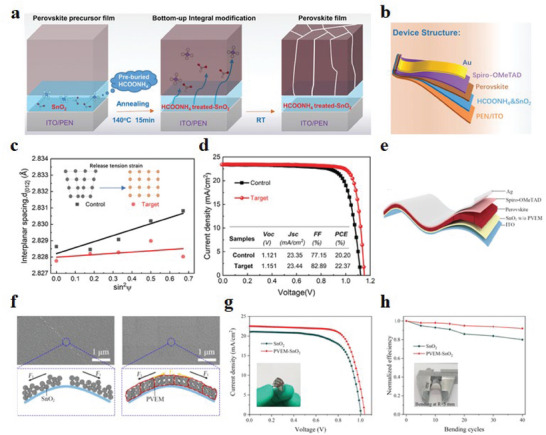
a) Schematic diagram of the preparation procedure of SnO_2_ ETL with HCOONH_4_ additive. b) f‐PSCs with PEN/ITO/HCOONH_4_ and SnO_2_/Perovskite/Spiro‐OMeTAD/Au structure. c) Lattice spacing d_(012)_ versus sin2Ψ plots for perovskite films formed on SnO_2_ ETLs without and with pre‐buried HCOONH_4_, respectively. d) The *J*–*V* curves and PV parameters (inset) of the champion f‐PSCs. Reproduced with permission.^[^ ^20^ ^]^ Copyright 2022, Wiley‐VCH. e) Schematic diagram of f‐PSCs with ITO/ SnO_2_ w/o PVEM/Perovskite/Spiro‐OMeTAD/Ag structure. f) SEM images of SnO_2_ and PVEM‐SnO_2_ and a schematic diagram of the internal state of the perovskite layer after being strained. g) The f‐PSCs *J*–*V* curves utilize pristine SnO_2_ and PVEM‐SnO_2_. h) Normalized PCE comparison of SnO_2_ and PVEM‐SnO_2_‐based f‐PSCs at a radius of 10 mm. Reproduced with permission.^[^ ^17^ ^]^ Copyright 2022, American Chemical Society.

**Figure 10:**
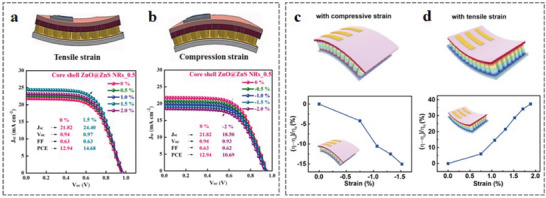
Schematic illustration of the deformation of an f‐PSC under a) tensile and b) compressive strain. *J*–*V* curves, PCE, and *J*_sc_ changes of f‐PSCs based on different strategies at 0%, 0.5%, 1%, 1.5%, and 2% tensile and compressive strain. Reproduced with permission.^[^ ^152^ ^]^ Copyright 2021, Elsevier. Schematic illustration of f‐PSCs deformation under c) compressive and d) tensile strains. The variation relationship of (*η*−*η*_0_)/*η*_0_ under continuous static compressive strain and tensile strain. Reproduced with permission.^[^ ^153^ ^]^ Copyright 2019, American Chemical Society.

As adjacent layers of the perovskite layer, the surface morphology and thickness of the ETL and HTL have an important influence on the fabrication of high‐quality perovskite films and the overall performance of the device.^[^ ^140^ ^]^ The strain state of the carrier transporting layer itself also has a significant impact on the device performance, and more and more researchers have been focusing on this aspect in recent years.^[^ ^141^, ^142^ ^]^ Zheng et al.^[^ ^20^ ^]^ used HCOONH_4_ as a pre‐embedded additive to the SnO_2_ ETL, and modified the ETL, the perovskite layer, and the interface between them through a top‐down infiltration process (**Figure** **9**a). The treatment of HCOONH_4_ not only enhanced the electron extraction ability of the ETL but also relaxed the microstrain and residual strain in the perovskite layer (Figure 9c). The f‐PSCs based on this strategy exhibited excellent photoelectric performance (Figure 9b), with a maximum PCE of 22.37%, and retained 90% of the initial PCE after 4000 bending cycles (Figure 9d). Since the development of a low‐temperature method to prepare SnO_2_ ETL in 2015, the PCE of SnO_2_‐based PSCs has increased to 25.8%.^[^ ^10^, ^143^, ^144^ ^]^ The characteristics of low‐temperature preparation of SnO_2_ can make it be applied in f‐PSCs. However, there have been some disadvantages of easy agglomeration and poor wettability in low‐temperature SnO_2_ for flexible application.^[^ ^145^, ^146^ ^]^ Qiu et al.^[^ ^17^ ^]^ incorporated the water‐soluble polymer PVEM into the SnO_2_ precursor solution to obtain a dense organic–inorganic hybrid PVEM‐SnO_2_ film, and based on this, f‐PSCs were fabricated (Figure 9e). The surface topography of SnO_2_ and PVEM‐SnO_2_ on the same substrate after ten bends is observed by scanning electron microscope (SEM) (Figure 9f). The target sample has no cracks and exhibits excellent bending stability. Combined with the stress map below the SEM image, it can be seen that the force between SnO_2_ and PVEM molecules offsets the stress caused by bending. After bending 40 times below 5 mm, the target device still maintained 92% of the initial PCE and exhibited superior electrical properties (Figure 9g,h). In piezoelectric optoelectronics, electrical and optical properties are coupled with piezoelectric properties. The strain induced by mechanical deformation induces polarized charges and changes the band structure at the interface.^[^ ^147^ ^]^ This method can be used to control the separation, transport, and combination of carriers to improve the photoelectric performance of photovoltaic devices.^[^ ^148^, ^149^ ^]^ In semiconductor devices, ZnO has excellent electron transport properties and is often used as an ETL material to study the piezoelectric photoelectron effect and enhance the performance of f‐PSCs. However, the Lewis basicity of ZnO and the surface defects in the film can lead to the degradation of the performance of the perovskite layer.^[^ ^150^, ^151^ ^]^ Fahim et al.^[^ ^152^ ^]^ used a hydrothermal method to grow ZnO nanorods on a flexible substrate, and sulfided the surface to generate ZnS, which passivated the defects of the perovskite film. Authors used this ETL to prepare f‐PSCs and apply different tensile and compressive strains by bending up or down to study the piezo‐phototronic effect. The study found that the photovoltaic parameters *V*_oc_ and *J*_sc_ were all increased under tensile strain. When the tensile strain was 1.5%, *J*_sc_ increased from 21.82 to 24.4 mA cm^−2^, and PCE increased from 12.84% to 14.68% (**Figure** **10**a). In contrast, the device performance decreased under compressive strain (Figure 10b). The applied tensile strain generates piezoelectric polarization charges in the ZnS layer, which regulates the energy level between the ZnS/perovskite layer and the ZnO/ZnO interface, thus significantly improving the PCE and mechanical stability of f‐PSCs. Similarly, Sun et al.^[^ ^153^ ^]^ used ZnO nanowires (NW) as an ETL for f‐PSCs and tested the piezoelectric potential distribution and optoelectronic properties of the devices at different strains, as well as the optoelectronic properties at different strains (Figure 10c,d). It has been found that the piezoelectric photoelectronic effect caused by applied tensile strain can enhance the performance of f‐PSCs. The performance enhancement of up to 40% at a tensile strain of 1.88% provides a new idea for enhancing device performance through external conditions (Figure 10d).

**Figure 11:**
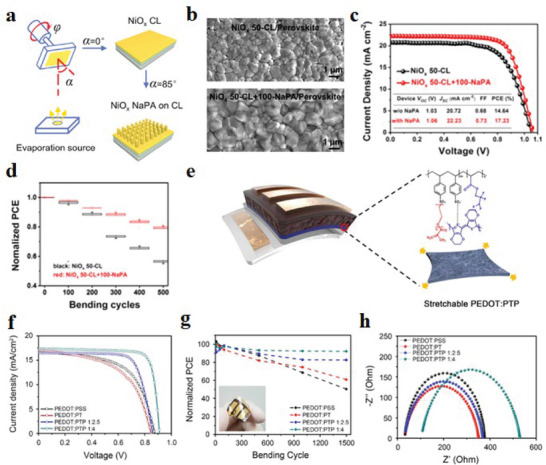
a) Demonstration images of NiO*_x_* compact layer and nanopillar array obtained with GLAD. b) SEM top‐down images (top, on the “50‐CL;” down, on the “50‐CL+100‐NaPA”). c) *J*–*V* curves of champion f‐PSCs. The inset is a comparative table of photovoltaic performance of champion f‐PSCs of “50‐CL” (black) and “50‐CL+100‐NaPA” (red). d) Distribution diagram of multiple measurement results of normalized PCE versus bending cycles of f‐PSCs. Reproduced with permission.^[^ ^155^ ^]^ Copyright 2019, American Chemical Society. e) Schematic diagram of the flexible device based on PEDOT:P(SS‐*co*‐TFPMA)‐*g*‐PEGMA HEL and the molecular structure of PEDOT:PTP. f) *J*–*V* properties of f‐PSCs containing solutions of different concentrations: PEDOT:PSS, PEDOT:P(SS‐*co*‐TFPMA), and PEDOT:P(SS‐*co*‐TFPMA)‐*g*‐PEGMA. g) Normalized PCE of the flexible device at a 7 mm bend radius. h) Nyquist plots of f‐PSCs under one sun illumination. Reproduced with permission.^[^ ^158^ ^]^ Copyright 2020, American Chemical Society.

**Figure 12:**
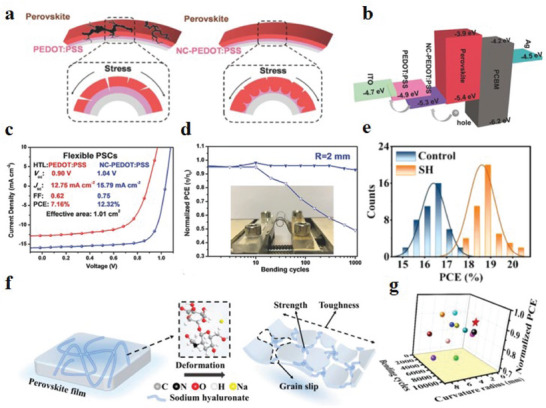
a) A stress relief scheme for perovskite layers based on NC‐PEDOT:PSS and PEDOT:PSS structures. b) Energy level diagram of PSCs. c) *J*–*V* curves and optoelectronic parameters of f‐PSCs based on PEDOT:PSS and NC‐PEDOT:PSS HTL. d) Normalized PCE bending cycle function of f‐PSCs at a bending radius of 2 mm (open and solid marks represent PEDOT:PSS HTL and NC‐PEDOT:PSS HTL, respectively). Reproduced with permission.^[^ ^159^ ^]^ Copyright 2017, Wiley‐VCH. e) Distribution of PCE statistics for 45 control and 45 SH‐based f‐PSCs devices. f) Schematic of SH acting on the perovskite film. g) The summary of normalized PCE and different bending conditions of recently reported f‐PSCs. Reproduced with permission.^[^ ^165^ ^]^ Copyright 2022, Wiley‐VCH.

In addition, NiO*_x_* is considered to be one of the ideal HTL materials because of its energy band suitable for perovskite and high hole mobility.^[^ ^154^ ^]^ Cong et al.^[^ ^155^ ^]^ successfully grown NiO*_x_* nanopillars vertically on ITO/PET flexible substrates using the GLAD method (**Figure** **11**a), and 100 nm NiO*_x_* nanopillar arrays (NiO*_x_* NaPAs) were deposited on the NiO*_x_* compact layer (NiO*_x_* CL) with a thickness of 50 nm as the HTL of f‐PSCs. It can be seen from SEM images that the NaPAs promote the nucleation and growth of perovskite grains, thus releasing the residual strain. (Figure 11b). Compared with the ordinary “50‐CL” device, the photoelectric performance of the f‐PSCs device based on “50‐CL+100NaPA” was significantly enhanced (*J*_sc_ was 22.23 mA cm^−2^, fill factor [FF] was 0.73%, and PCE was 17.23%) (Figure 11c). The addition of “100NaPA” is beneficial to enhance the carrier transport ability and light absorption ability of the perovskite layer, and the stress and residual strain can be released due to the gaps between NiO*_x_* NaPAs. The targeted f‐PSCs have good flexibility and still maintain more than 80% of their initial PCE after 500 bending cycles (Figure 11d). PEDOT:PSS has high transparency and mechanical stability, which is one of the commonly used HTL layers in inverted devices.^[^ ^156^, ^157^ ^]^ However, the traditional PEDOT:PSS exhibits a low tensile strain fatigue limit, leading to the destruction of the surface morphology and loss of conductivity when the films are flexed. Rhee et al.^[^ ^158^ ^]^ successfully synthesized PEDOT:P(SS‐*co*‐TFPMA)‐*g*‐PEGMA(PEDOT:PTP) copolymer ionomer, and it is used as the HTL of the inverted f‐PSCs (Figure 11e). PEDOT:PTP 1:4 film has excellent stretchability and will not break under 300% tensile strain. The conductivity after stretching remains above 60%, making it an ideal HTL material for f‐PSCs. The f‐PSCs based on this material have better *V*_oc_ and *J*_sc_ than conventional devices. The target device still maintained 92% of its initial PCE after bending 1500 times, showing excellent stability (Figure 11g). Further studying the carrier transport and recombination kinetics of the target device by Nyquist plots, the recombination of PEDOT:PTP 1:4 is reduced and the electrical resistance is slightly increased, benefiting from the reduction of residual strain (Figure 11h). Hu et al.^[^ ^159^ ^]^ prepared a polystyrene‐doped nanocellular PEDOT:PSS HTL (NC‐PEDOT: PSS) through a low‐temperature process. This material not only improves the light utilization efficiency of the device but also plays a supporting role. The nanocellular scaffold relieves the mechanical stress during bending, thus it improves the flexibility and stability of the device (**Figure** **12**a). The energy level diagram of PSCs illustrates that nanocellular PEDOT:PSS (NC‐PEDOT:PSS) HTL effectively facilitates the extraction of charge carriers (Figure 12b). Compared with the control device, the f‐PSCs device based on this structure has greatly improved photoelectric properties and PCE. *V*_oc_ increased from 0.9 to 1.04 V, *J*_sc_ increased from 12.75 to 15.79 mA cm^−2^, FF increased from 0.62% to 0.75%, and PCE increased from 7.16% to 12.32% (Figure 12c). After 1000 bending cycles at 2 mm, 93% of the initial PCE was maintained (Figure 12d).

#### 5.2.3 Light Absorbing Layer

**Figure 13:**
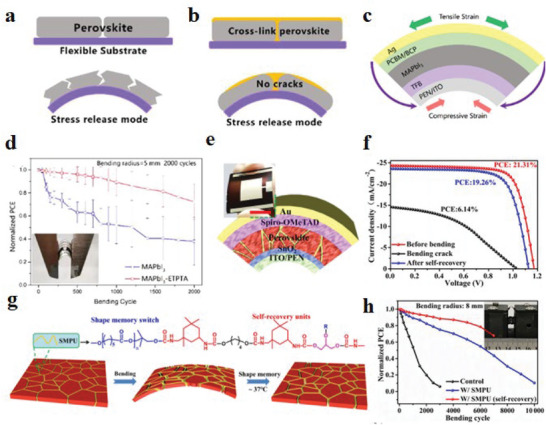
a,b) Schematic diagram of the fracture states of f‐PSCs based on different strategies after being subjected to bending strain. c) Schematic diagram of the f‐PSCs devices structure in the bent state. d) Normalized PCE cycle function of the f‐PSCs at a bending radius of 5 mm. Reproduced with permission.^[^ ^166^ ^]^ Copyright 2021, Elsevier. e) Device structure composition of f‐PSCs. The inset photo is a large‐area f‐PSCs. f) Bend recovery performance of devices with SMPU. g) The action process and principle of SMPU at the GBs of perovskite thin films. h) Normalized average PCE of f‐PSCs as a function of bending cycle with a bending radius of 8 mm. Reproduced with permission.^[^ ^115^ ^]^ Copyright 2022, Wiley‐VCH.

**Figure 14:**
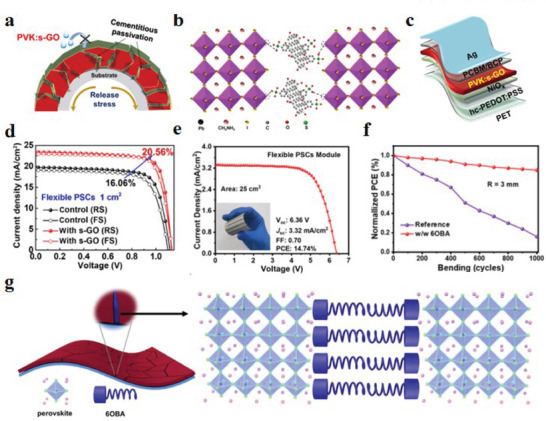
a) Schematic diagram of s‐GO cementing and passivating the GBs of the perovskite layer to block moisture attack and improve toughness. b) Microscopic demonstration of the interaction between s‐GO and perovskite layers. c) Configuration of the f‐PSCs. d) *J*–*V* curves of the best‐performing flexible device with or without s‐GO measured in the reverse and forward scan directions. Reproduced with permission.^[^ ^173^ ^]^ Copyright 2020, Elsevier. e) *J*–*V* curves and other optoelectronic parameters of flexible devices based on 6OBA perovskite thin films. f) Normalized average PCE of f‐PSCs as a function of the bending cycles with a radius of 3 mm. g) Schematic diagram of stress release of perovskite film with 6OBA and microscopic view of the interaction between 6OBA and perovskite film. Reproduced with permission.^[^ ^68^ ^]^ Copyright 2022, Wiley‐VCH.

The effect of intrinsic strain on f‐PSCs mainly occurs during the formation of the perovskite layer. According to related reports, the methods for improving the strain of the perovskite layer mainly include the selection of A‐site cations in the perovskite composition, the addition of additives to the perovskite precursor solution, and the addition of additives to the anti‐solvent solution.^[^ ^160^, ^161^, ^162^ ^]^ The coordination of different A‐site cations can regulate the lattice contraction to release the strain to improve the performance and stability of f‐PSCs. In addition, the additives in the precursor can adjust the strain without changing the lattice, and the additives in the anti‐solvent can adjust the strain of the upper layer of the perovskite. For optoelectronic materials, it is difficult to resolve the contradiction between toughness and strength while maintaining high optoelectronic properties.^[^ ^163^, ^164^ ^]^ Lu et al.^[^ ^165^ ^]^ designed a network topology that simultaneously improved the strength and flexibility of perovskite films. The designed sodium hyaluronate (SH) hydrogel is composed of a strong polymer network and is highly elastic. Its functional groups interact strongly with the perovskite material, which can release the strain of the perovskite film and prevent the grain from slipping (Figure 12f). The introduction of SH significantly improved the flexibility of the perovskite film, the elongation increased from 1.58% to 5.02%, and the fracture strength increased from 23.13 to 55.25 MPa. The inverted f‐PSC based on this material still maintained 90% of the initial PCE after bending 6000 times below 2 mm and achieved a champion PCE of 20.01% (Figure 12e,g). When stress is applied to the perovskite film, cracks first appear at the GBs, and then propagate along the GBs network to the edge. However, the mechanical fracture of GBs is difficult to heal itself, which affects the stability of the device.^[^ ^23^ ^]^ Guo et al.^[^ ^166^ ^]^ added a cross‐linked ethoxylated trimethylolpropane triacrylate (ETPTA) to the perovskite precursor solution. During perovskite nucleation and crystallization, ETPTA can fill GBs and voids to form dynamic elastic GBs, thereby repairing strain‐induced cracks (**Figure** **13**a,b). Based on this strategy (Figure 13d), self‐healing polymer materials for GB cracks in perovskite thin films have also received extensive attention.^[^ ^167^, ^168^, ^169^ ^]^ Xue et al.^[^ ^115^ ^]^ reported a method for real‐time self‐healing of GB cracks in f‐PSCs using SMPU (Figure 13g). The SMPU molecules are evenly distributed at the GBs of the perovskite film, which not only passivates the defects at the GBs, releases mechanical stress, but also repairs GBs cracks at a temperature of 37 °C. The f‐PSCs prepared based on this method achieved a high PCE of 21.33% (Figure 13e,f), and maintained 80% of the initial PCE after 6000 cycles of 8 mm bending (Figure 13h). In the bending state, due to the influence of stress and strain, GBs are the most important locations for cracks in the perovskite film, and moisture will also corrode the perovskite film through GBs, reducing the environmental stability of the device.^[^ ^170^, ^171^, ^172^ ^]^ Inspired by concrete, Hu et al.^[^ ^173^ ^]^ developed a method to construct gelled GBs using sulfonated graphene oxide (s‐GO). The sulfonic acid groups in s‐GO strongly interact with the lead atoms in the perovskite film, enhancing the toughness, ductility, and hydrophobicity of GBs, and releasing the strain (**Figure** **14**a,b). The highest PCE of f‐PSCs prepared based on this layer (Figure 14c) is 20.56%, *J*_sc_ is 23.44 mA cm^−2^, *V*_oc_ is 1.12 V, and the *J*–*V* hysteresis is weakened (Figure 14d,e).

**Figure 15:**
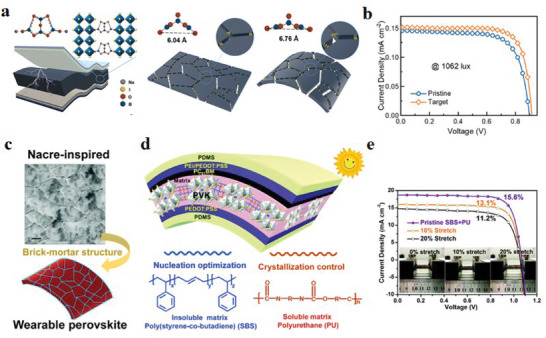
a) Schematic diagram of f‐PSCs with a structure of PEN/ITO/SnO_2_/Perovskite/Spiro‐OMeTAD/MoO_3_/Ag and a schematic diagram of the structure of borax and the mechanism of interaction between borax and perovskite at the GBs. Schematic before and after bending of a flexible perovskite film with borax. b) *J*–*V* curves of f‐PSCs before and after borax treatment. Reproduced with permission.^[^ ^54^ ^]^ Copyright 2022, Wiley‐VCH. c) Schematic diagram of a perovskite film with a brick‐concrete structure. The top inset is the SEM image of nacre. d) Structural composition of wearable PSCs and chemical structures of insoluble SBS and soluble PU. e) *J*–*V* curves of champion f‐PSCs obtained at 0%, 10%, and 20% tensile strain. Reproduced with permission.^[^ ^181^ ^]^ Copyright 2019, The Royal Society of Chemistry.

Perovskite solute uniformity and crystal orientation have been shown to affect the crystallization of printed perovskite films.^[^ ^174^, ^175^ ^]^ Considering the benefits of temperature‐sensitive liquid crystals for the printing and crystallization of shrinkable perovskite films, Yang et al.^[^ ^73^ ^]^ introduced 6OBA into the perovskite precursor solution to print f‐PSCs on a large scale. 6OBA mainly exists at the GBs of perovskite films, and its own elastic chain can promote the release of residual stress at the GBs (Figure 14g). Based on this strategy to obtain f‐PSCs with excellent performance, six individual cells are connected in series to form 25 cm^2^ f‐PSCs, with a PCE as high as 14.74% and excellent mechanical stability (Figure 14f). Inspired by balloon glue, Chen et al.^[^ ^54^ ^]^ spin‐coated borax on the pre‐crystallized perovskite film to achieve GB penetration, and obtained high‐quality perovskite film. The oxygen ions on both sides of borax molecules can form strong coordination bonds with lead ions and play a certain role in stretching bridges. Through molecular vibration model calculations, it is found that the stretching ratio of borax molecules and oxygen ions on both sides can reach 12%, and the perovskite film can withstand greater strain (**Figure** **15**a). The f‐PSCs based on this strategy exhibited better *V*_oc_ and *J*_cs_ than the control one, and the PCE was 21.63% (Figure 15b).

**Figure 16:**
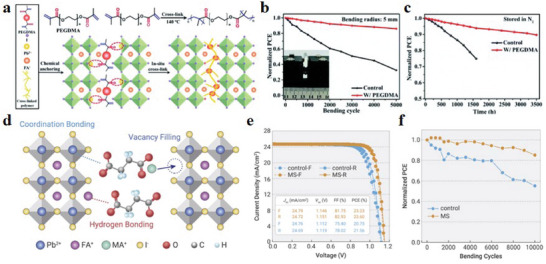
a) Schematic illustration of the working mechanism of PEGDMA in f‐PSCs. b) The relationship between normalized PCE and bending period at a bending radius of 5 mm in a f‐PSCs device. c) Long‐term stability of f‐PSCs under N_2_ atmosphere. Reproduced with permission.^[^ ^114^ ^]^ Copyright 2022, The Royal Society of Chemistry. d) Schematic illustration of the interaction between perovskite grains and MS molecules at the GBs. e) *J*–*V* curves and photovoltaic performance parameters of champion f‐PSCs. f) Stability of f‐PSCs at a bending radius of 6 mm. Reproduced with permission.^[^ ^53^ ^]^ Copyright 2022, Elsevier.

It is difficult for f‐PSCs to adapt to complex limb movements such as bending, stretching, folding, and twisting.^[^ ^176^, ^177^ ^]^ The insoluble matrix in the nacre structure acts as a framework structure, and the soluble matrix controls the directional growth of crystals, which has been used to release the strain in some flexible devices.^[^ ^178^, ^179^ ^]^ Inspired by this structure, Hu et al.^[^ ^180^ ^]^ adopted a biomimetic crystallization method to introduce insoluble poly(styrene‐*co*‐butadiene) (SBS) and soluble polyurethane (PU) into the perovskite layer. The two additives produce a synergistic effect that improves the mechanical properties of the f‐PSCs (Figure 15c). Specifically, the SBS scaffold reduces the number of nucleation sites and promotes heterogeneous nucleation, and PU inhibits the crystallization rate of perovskite crystals and together promotes stress release, resulting in high‐quality perovskite films (Figure 15d). Devices prepared on the basis of this structure remained highly stable in the stretched state, with a PCE of 7.91% for a large area of 56.02 cm^2^ of flexible devices (Figure 15e). The addition of hydroxyl and carboxyl polymers as scaffolds improved the stability of the f‐PSCs.^[^ ^169^, ^181^ ^]^ However, solution precipitation in the precursors was unavoidable due to the strong interaction between the polymers and PbI_2_. Xue et al.^[^ ^114^ ^]^ used the macromer PEGDMA to in situ cross‐link perovskite crystals to form a network polymer (**Figure** **16**a), which effectively regulated the crystallization process of the perovskite film during annealing and released the residual strain. The PCE of the f‐PSCs prepared based on this strategy is as high as 21.41%, and it still maintains 86% of the initial PCE after 5000 times of bending (5 mm) (Figure 16b), and it also has a long‐term stability of 3500 h under N_2_ atmosphere (Figure 16c). The microstrain originates from the disorder and defects of the local lattice, and the modification of the doped perovskite lattice with cations such as Cd^2+^ and MDA^2+^ can reduce the microstrain, according to the report.^[^ ^69^, ^182^ ^]^ Li et al.^[^ ^53^ ^]^ incorporated bilateral MS molecules into the perovskite film to alleviate the strain and interface defects in the perovskite film. The two terminal hydroxyl groups in MS can form hydrogen bonds with the formamidines of two adjacent perovskite grains, which can release the strain caused by the environment (Figure 16d). The f‐PSCs based on this method have *J*_sc_ of 24.72 mA cm^−2^, *V*_oc_ of 1.15 V, FF of 82.93%, and a PCE of 23.6% (Figure 16e). After 10 000 bending cycles at 6 mm, 85% of the initial PCE is still maintained (Figure 16f).

**Figure 17:**
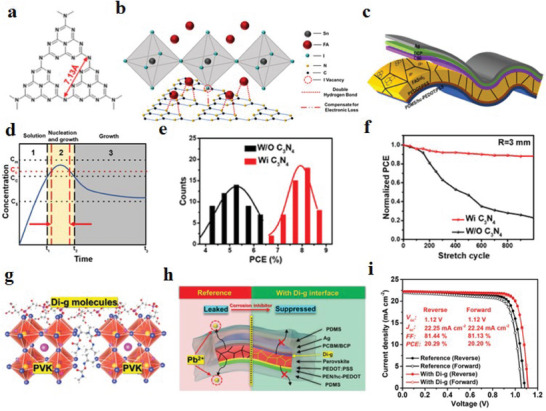
a) Chemical structural unit of g‐C_3_N_4_. b) Schematic illustration of the interaction between g‐C_3_N_4_ and perovskite thin films (including passivation and bonding). c) f‐PSCs with a structural composition of PDMS/hc‐PEDOT:PSS/PE‐DOT‐PSS/FASnI_3_/C60/BCP/Ag. d) The concentration change during crystal nucleation and growth is represented according to the Lamer curve. e) PCE histograms of 50 reference devices and 50 g‐C_3_N_4_ devices. f) Normalized PCE periodic function of f‐PSCs at a bending radius of 3 mm. Reproduced with permission.^[^ ^185^ ^]^ Copyright 2021, Wiley‐VCH. g) Schematic diagram of the interaction mechanism between Di‐g and perovskite grains. h) Composition structure of f‐PSCs device. i) *J*–*V* curves and photovoltaic data measured in reverse and forward directions for f‐PSCs with or without Di‐g. Reproduced with permission.^[^ ^16^ ^]^ Copyright 2021, Wiley‐VCH.

Compared with lead‐based perovskites, Sn‐based perovskites have the characteristics of less toxicity and better mechanical bending properties. However, there is still a huge gap in PCE and stability, which is manifested in the poor crystallinity of perovskite films and the easy oxidation of Sn^2+^.^[^ ^183^, ^184^ ^]^ Inspired by the grain size effect of antifreeze proteins, Rao et al.^[^ ^185^ ^]^ introduced 2D graphite phase C_3_N_4_ (g‐C_3_N_4_) (**Figure** **17**a) into the Sn‐based perovskite layer to optimize the nucleation and crystallization process, effectively passivating surface defects and improving strain distribution (Figure 17b,d). The inverted f‐PSCs fabricated based on this method have excellent reliability (Figure 17c). The PCE distribution is narrow, with more than 80% of the devices distributed above 7.41% (Figure 17e). The device combined with g‐C_3_N_4_ still maintains 90% of the initial PCE after 600 bending cycles (Figure 17f), g‐C_3_N_4_ eliminates the lattice distortion caused by residual stress and improves the stability of the flexible device.

### 5.3 Interface Engineering

**Figure 18:**
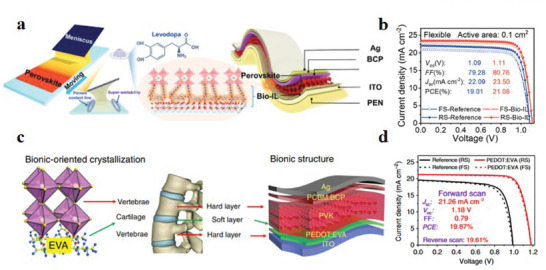
a) Schematic diagram of the preparation process of perovskite thin films by meniscus method, biomimetic mechanism, and structure of f‐PSCs. b) *J*–*V* curves and photovoltaic performance of small‐area (0.1 cm^2^) f‐PSCs measured in forward and reverse scans. Reproduced with permission.^[^ ^195^ ^]^ Copyright 2022, Wiley‐VCH. c) Vertebral physiology and biomimetic mechanism and device composition of f‐PSCs. d) *J*–*V* curves and photovoltaic performance of f‐PSCs measured in reverse and forward directions. Reproduced with permission.^[^ ^19^ ^]^ Copyright 2020, Nature Publishing Group.

**Figure 19:**
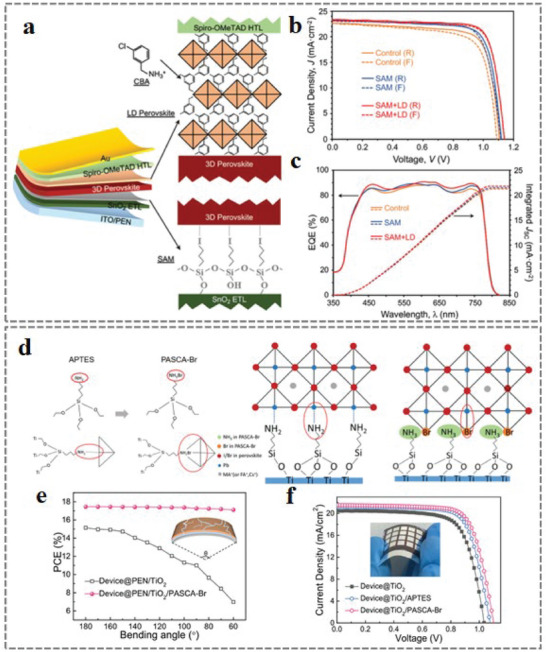
a) Schematic diagram of the composition structure and principle of the dual‐interface enhanced flexible device. b) Forward and reverse scan *J*–*V* curves of control, SAM, and SAM+LD f‐PSCs. c) EQE and *J*_sc_ spectra of the champion flexible devices of control, SAM, and SAM+LD. Reproduced with permission.^[^ ^196^ ^]^ Copyright 2022, Wiley‐VCH. d) Protonated amino terminals (R‐NH_3_Br) and lattice structure of 3‐aminopropyl triethoxysilane (APTES) and PASCA‐Br modified interfaces. e) Variation curves of PCE of flexible devices with or without PASCA‐Br under different bending angles. The inset is a schematic diagram of the bending change simulation. f) *J*–*V* curves of f‐PSCs based on TiO_2_, TiO_2_/APTES, and TiO_2_/PASCA‐Br strategies. The illustration is a schematic diagram of the actual product. Reproduced with permission.^[^ ^83^ ^]^ Copyright 2022, Wiley‐VCH.

In f‐PSCs, there are problems of insufficient adhesion of perovskite layers and fragility at the contact interface.^[^ ^51^, ^186^, ^187^ ^]^ These inherent defects reduce the stress tolerance and mechanical stability of photovoltaic devices. There have been reports of such problems being solved through interface engineering methods such as self‐assembling middle layers and layered interfaces.^[^ ^51^, ^186^ ^]^ Some excellent intermediate layers can release the residual strain between the perovskite layer and the critical layer (ETL/perovskite, perovskite/HTL) to improve device performance and stability.^[^ ^188^, ^189^ ^]^ On account of the migration of iodide ions and the volatilization of methylamine ions in the perovskite crystal, the leakage of lead ions will occur due to the diffusion effect.^[^ ^190^, ^191^ ^]^ The introduction of a high‐quality interlayer between the critical layers of perovskite films is one of the effective methods to inhibit the diffusion of toxic ions and protect perovskite films, and leakage of lead will have certain negative impacts on the environment and the human body, so it is necessary to improve the moisture resistance and oxygen resistance of perovskite devices and suppress lattice degradation from the perspective of lattice stress.^[^ ^192^, ^193^ ^]^ Inspired by the non‐selective permeability of inactivated cell membranes, Meng et al.^[^ ^16^ ^]^ introduced diphosphatidyl‐glycerol (Di‐g) as an interface layer to modify the surface of perovskite films. Di‐g molecules are anchored on the lattice structure of the surface of the perovskite layer, which can release the residual stress and improve the water‐resistance of the perovskite film. Thereby this strategy can prevent device performance degradation and lead leakage (Figure 17g). The f‐PSCs prepared based on this strategy exhibited better performance than unmodified devices (Figure 17h). The PCE increased from 18.80% to 20.29%, the *V*_oc_ was 1.12 V, the *J*_sc_ was 22.25 mA cm^−2^, and the FF was 81.44%, without hysteresis effect (Figure 17i). During the meniscus printing process, due to the existence of the coffee ring effect, defects, such as pinholes and poor interface contact, will occur during the film formation of perovskite films. This will result in reduced strength and strain generation.^[^ ^194^ ^]^ Inspired by the bio‐glue of barnacles, Fan et al.^[^ ^195^ ^]^ introduced a levodopa biomimetic interfacial layer (Bio‐IL) in f‐PSCs. This layer suppresses the coffee ring effect during meniscus printing. The viscous coordination of Bio‐IL and lead ions improves the quality of the perovskite film, releases the residual strain, and promotes the development of large‐area optoelectronic devices (**Figure** **18**a). A small‐area (0.1 cm^2^) f‐PSCs based on this strategy has a reverse scanning champion PCE of 21.08% (Figure 18b). Meng et al.^[^ ^19^ ^]^ synthesized PEDOT:EVA by a miniemulsion method as an HTL between ITO and perovskite films to promote vertical crystallization of perovskite films and improve device flexibility (Figure 18c). Compared with the control group, the *V*_oc_ of f‐PSCs obtained by this method was 1.18 V, the PCE was 19.87%, and the initial efficiency of 85% was maintained after 7000 bending cycles (Figure 18d). In earlier f‐PSCs studies, only the interface between 3D‐(metal halide perovskite) MHP and HTL was strengthened using an overlay of LD MHP grown in situ on 3D‐MHP. In addition, the use of self‐assembled monolayers (SAMs) can strengthen the interface between ETL and 3D‐MHP. However, both interfaces need to be enhanced simultaneously to produce synergistic beneficial effects. Dai et al.^[^ ^196^ ^]^ simultaneously used SAM and LD‐MHP to enhance the ETL/3D‐MHP interface and 3D‐MHP/HTL interface. Careful toughness measurements and detailed mechanical modeling demonstrate that the dual interface enhances the interfacial mechanical properties of f‐PSCs (**Figure** **19**a). In comparison with the photovoltaic parameters of the control group, the target device shows high performance with a *J*_sc_ of 23.39 mA cm^−2^, *V*_oc_ of 1.138 V, and PCE of 21.03% (Figure 19b,c).

**Figure 20:**
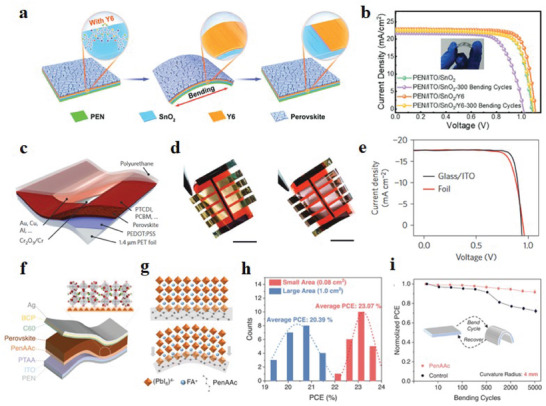
a) Schematic diagram of the mechanism of action of Y6. b) *J*–*V* curves of f‐PSCs with or without Y6 and after 300 bending cycles, respectively. Reproduced with permission.^[^ ^198^ ^]^ Copyright 2022, Wiley‐VCH. c) Schematic diagram of the structural composition of f‐PSCs. Freestanding 3‐micron thick solar cell with gold top metal. Perovskite solar foils with low‐cost copper back contacts. d) *J*–*V* scanning of flexible devices under simulated solar illumination. e) *J*–*V* scanning of flexible devices under simulated solar illumination. Reproduced with permission.^[^ ^11^ ^]^ Copyright 2022, Nature Publishing Group. f) Schematic diagram of the f‐PSCs structure. g) Schematic diagram of interfacial stress release after Pentylammonium Acetate (PenAAc) treatment. h) Histograms of PCE values of 22 devices for small‐area (0.08 cm^2^) and large‐area (1 cm^2^) f‐PSCs, respectively. i) Efficiency of f‐PSCs (0.08 cm^2^) at a radius of curvature of 4 mm as a function of mechanical bending cycles. Reproduced with permission.^[^ ^199^ ^]^ Copyright 2023, Wiley‐VCH.

Interfacial strain and lattice distortion are unavoidable during the crystallization of perovskite thin films.^[^ ^107^ ^]^ SAM and some organics with rich functional groups act as passivation interface layers and form coordination between functional layers to improve stress distribution in perovskite films. Zhang et al.^[^ ^83^ ^]^ introduced a protonated amine silane coupling agent (PASCA‐Br) between the ETL and the perovskite layer. The PASCA‐Br interlayer can firmly sandwich the TiO_2_ and perovskite crystals, releasing the lattice stress on the perovskite side, thereby suppressing the interfacial strain and enhancing the stability of the f‐PSCs (Figure 19d). The f‐PSCs have excellent bending resistance, maintaining 98% of the initial PCE at 60° bending with minimal fluctuations in PV parameters (Figure 19e). The flexible device exhibited optoelectronic performance with *V*_oc_ of 1.10 V, *J*_sc_ of 21.54 mA cm^−2^, and a maximum PCE of 17.45% (Figure 19f). During f‐PSCs bending, SnO_2_ ETL undergoes significant cracking, generating large residual stresses and resulting in degradation of device performance.^[^ ^197^ ^]^ Liu et al.^[^ ^198^ ^]^ introduced a semi‐planar organic non‐fullerene small molecule BT‐core‐based fused‐unit dithienothiophen[3,2‐b]‐pyrrolobenzothiadiazole derivative (Y6) between SnO_2_ and the perovskite layer as a buffer layer to release the residual stress generated during annealing and reduce the formation of defects during bending (**Figure** **20**a). The f‐PSCs based on SnO_2_/Y6 substrate exhibit excellent optoelectronic performance, with a PCE of 20.09% (Figure 20b). The device performance dropped to 18.42% after 300 bending cycles at a radius of 8 mm, showing good mechanical stability.

The judicious selection of HTLs, ETLs, and contact metals, sandwiching active layers of alkylammonium lead trihalide perovskites (RNH_3_PbX_3_) is a way to maximize performance and stability. Kaltenbrunner et al.^[^ ^11^ ^]^ introduced a chromium oxide/chromium interlayer on the perovskite film, which effectively blocked the erosion of water and oxygen, extended the life of the device, and could withstand 50% linear compression (Figure 20c). *V*_oc_ and FF barely change upon linear compression, but the folds and wrinkles generated during bending act as light‐harvesting microstructures, enhancing the light absorption capability of the device. (Figure 20d,e). Since GBs are the most prone to cracks under external strain, the passivation of grain boundary defects is becoming increasingly important in f‐PSCs. Gao et al.^[^ ^199^ ^]^ found that PenA^+^ and Ac^−^ had a strong chemical combination with the surface of the perovskite film through the synthesis and regulation of anions and cations. Pentylammonium Acetate (Pen Acc) was introduced at the perovskite/HTL interface to enhance the ability to resist strain and reduce the surface defects of the perovskite layer (Figure 20f,g). A small‐area (0.08 cm^2^) inverted f‐PSCs obtained based on this strategy has a high PCE of 23.35% and still maintains 91% of the initial PCE after bending 5000 times (Figure 20h,i).

## 6 Summary and Perspective

### 6.1 Summary

Strain has become an indispensable research object in high‐performance and high‐stability f‐PSCs. Through the objective analysis of the source of strain, related research explored strain simulation and characterization methods, explored the influence of strain on the mechanism of f‐PSCs, and then proposed a method to control/use strain to improve the photoelectric performance of the device.

The effects of strain on the physical properties of f‐PSCs mainly include carrier transport, band gap, defect density, and non‐radiative recombination. The carrier mobility increases with increasing compressive strain, but after a certain point, if the compressive strain continues to increase, dislocation occurs and the carrier mobility decreases. In addition, compressive strain enhances the overlap of atomic orbitals, and increases the charge transport rate and conductivity, while tensile strain does the opposite. The band gap is positively correlated with tensile strain and negatively correlated with compressive strain, and narrows with increasing compressive strain. Notably, strain affects the nucleation process of perovskite films, which in turn affects the grain size, uniformity, defect density, and nonradiative recombination.

In testing the mechanical properties of f‐PSCs devices, in addition to quantitative analysis and calculation, XRD/GIXRD, Raman spectroscopy, TEM, PL, and TRPL can also be used to characterize the strain indirectly. With the development of technology, it is now possible to analyze the strain state in devices by FEM such as the Derjaguin–Muller–Toporov (DMT) model.

We summarize the methods to control/utilize the strain to enhance the optoelectronic performance and stability of f‐PSCs. Due to the difference in thermal expansion coefficient, the optimization of substrate material can effectively control the strain of the perovskite layer. By adjusting the manufacturing process of flexible devices, we can reduce the lattice strain generated in the crystal formation process such as reducing the annealing temperature, growing perovskite films by low‐temperature methods, and using new processes to obtain different functional layers. Furthermore, we can develop new processes to obtain high‐performance ETL and HTL or use cationic alloying methods to obtain high‐quality, low‐strain perovskite layers. Interface engineering that introduces a new molecule between the perovskite layer and its adjacent layer is also an important way to regulate strain during bending and device fabrication.

Through the study of strain in f‐PSCs, high‐efficiency and stable devices are realized to promote the development of their applications. The flexibility of f‐PSCs endows them with a wider range of applications than rigid PSCs including wearable electronics, clean energy vehicles, power plants for aircraft and aerospace, and smart buildings. Combining energy storage and electronics can enable wearable functional electronic systems including smart watches and glasses, motion sensors, and smart displays. The development of transparent electrodes facilitates the realization of semitransparent f‐PSCs, which are expected to realize their value in smart displays and windows. Combined with energy storage devices, f‐PSCs can provide a source of electricity. For example, flexible self‐charging perovskite solar capacitors composed of f‐PSCs and capacitors can simultaneously harvest and store energy. In addition, possible future applications can be envisioned. For example, f‐PSCs‐fuel conversion system, photo/electrocatalytic system based on f‐PSCs power supply, integrated water splitting system based on f‐PSCs, and integrated system for carbon dioxide emission reduction based on f‐PSCs.

### 6.2 Perspective

It has been proved that the PCE and stability of f‐PSCs can be significantly improved by adjusting and analyzing the strain of f‐PSCs. We propose the future challenges and prospects of strain engineering in f‐PSCs. It is well known that parameters, such as *J*_sc_, *V*_oc_, and FF, which are important indicators for evaluating PCE performance, can be effectively improved by eliminating surface defects in the active layer, increasing carrier mobility, and reducing non‐radiative compounding. The enhancement of these photoelectric parameters will be effectively manipulated thus increase in PCE. The octahedral BX_6_^4−^ unit in the perovskite structure has been quite well studied, and the relationship between octahedral tilt and strain has also been noted. However, the relationship between the bond angle B─X─B and key physical features such as the band gap remains to be clarified, and it is significant to explore a method to clearly express this structural feature, and there are few quantitative calculation and analysis methods, that are worthy of further study.

The inhomogeneous spatial distribution of residual stress in the perovskite film can lead to the accumulation of elastic energy, which will be attacked by water and oxygen. The stress at the GBs will bring some hidden interface fracture and thus reduce the stability of the device. Determining the distribution and variation regulations of stress is an effective way to solve the above problems and an indispensable method to obtain ideal devices. Efforts should be made to make the perovskite film in the neutral layer state, which is more conducive to the realization of stable and efficient f‐PSCs and is also more conducive to its commercial application. In addition, the perovskite film is in a certain compressive strain state, which can enhance the charge transport rate and electrical conductivity, and improve the absorbance. The perovskite film is in a certain tensile strain state, which can prolong the PL lifetime, expand the bandgap, and adjust the energy level. Therefore, more innovative strategies should be developed to exploit the advantages of perovskite films under different strains to achieve higher‐performance f‐PSCs. The arrangement of the components in the alloy system (mixed cation and halide system) remains to be clarified such as whether it varies from unit cell to unit cell. In the case of mixed halide samples, the observed strain may be due to vibration of composition and a tendency to form clusters. Nanoscale chemical mapping techniques are needed to improve the understanding of chemical components. In addition, in situ strain detection is required to view the propagation of strain in real time. The relevant paper has begun to explore the strain in the out‐of‐plane direction, but the principle of the important influence of out‐of‐plane strain on the perovskite remains to be clarified, thus more in‐depth detection technology is needed.

An in‐depth understanding of the strain in f‐PSCs is necessary for the pursuit of ideal flexible devices and the early realization of the commercial application. Most analyses of strain in f‐PSCs have focused on the perovskite layer, but less research has been done on the interaction between the perovskite layer and adjacent layers. It is therefore important to develop characterization techniques that characterize interlayers and even the entire device to gain a comprehensive understanding of strain. As far as the existing methods are concerned, it is necessary to develop new or improved processes for obtaining high‐quality perovskite films, explore new additives and interface materials, and develop new flexible substrates to regulate strain in flexible devices. The ion migration phenomenon in perovskite films seriously affects the stability of f‐PSCs. Therefore, it is necessary to develop more advanced techniques to quantitatively analyze the relationship between the strain relaxation effect and ion migration, so as to provide a better direction for the preparation of high‐quality perovskite thin films. Furthermore, the measurement method for the stability of flexible devices should be closer to the actual application. For example, in the actual application of wearable devices, in addition to bending, there are also twisting and wrinkling. More importantly, after f‐PSCs work for a period of time, there are few studies on the creep effect in perovskite films. Means for indirect or direct characterization of torque can be developed, which is believed to advance practical commercial applications. The suitable bandgap and bendable properties of f‐PSCs make them have a wide range of applications, and their advantages can be used to design more advanced, intelligent, and commercially friendly products.

## Conflict of Interest

The authors declare no conflict of interest.
